# An ionic lock and a hydrophobic zipper mediate the coupling between an insect pheromone receptor BmOR3 and downstream effectors

**DOI:** 10.1016/j.jbc.2021.101160

**Published:** 2021-09-02

**Authors:** Jing-Yu Lin, Zhao Yang, Chan Yang, Ji-Xiang Du, Fan Yang, Jie Cheng, Wei Pan, Shi-Jie Zhang, Xu Yan, Jia Wang, Jin Wang, Lu Tie, Xiao Yu, Xin Chen, Jin-Peng Sun

**Affiliations:** 1 Key Laboratory Experimental Teratology of the Ministry of Education and Department of Physiology, School of Basic Medical Sciences, Shandong University, Jinan, China; 2 Key Laboratory Experimental Teratology of the Ministry of Education and Department of Biochemistry and Molecular Biology, School of Basic Medical Sciences, Shandong University, Jinan, China; 3 Department of Physiology and Pathophysiology, School of Basic Medical Sciences, Peking University, Key Laboratory of Molecular Cardiovascular Science, Ministry of Education, Beijing, China; 4 Department of Pharmacology, School of Basic Medical Sciences, Shandong University, Jinan, China; 5 Department of Pharmacology, School of Basic Medical Sciences, Peking University, Beijing, China; 6 Department of Medicinal Chemistry, School of Pharmaceutical Engineering and Life Science, Changzhou University, Changzhou, Jiangsu, China

**Keywords:** *Bombyx mori*, receptor, BmOR3, BmOrco, calcium channel, structure–function, G protein, arrestin, FlAsH-BRET, fluorescein arsenical helix–bioluminescence resonance energy transfer, GPCRs, G protein-coupled receptors, HEK293, human embryonic kidney 293, ICLs, intracellular loops, ORs, olfactory receptors, OSNs, olfactory sensory neurons, PR, pheromone receptor, PTX, pertussis toxin, S7, segment 7, *trans*-7TM, transverse seven transmembrane, β2AR, β2 adrenoreceptor

## Abstract

Pheromone receptors (PRs) recognize specific pheromone compounds to guide the behavioral outputs of insects, which are the most diverse group of animals on earth. The activation of PRs is known to couple to the calcium permeability of their coreceptor (Orco) or putatively with G proteins; however, the underlying mechanisms of this process are not yet fully understood. Moreover, whether this transverse seven transmembrane domain (7TM)-containing receptor is able to couple to arrestin, a common effector for many conventional 7TM receptors, is unknown. Herein, using the PR BmOR3 from the silk moth *Bombyx mori* and its coreceptor BmOrco as a template, we revealed that an agonist-induced conformational change of BmOR3 was transmitted to BmOrco through transmembrane segment 7 from both receptors, resulting in the activation of BmOrco. Key interactions, including an ionic lock and a hydrophobic zipper, are essential in mediating the functional coupling between BmOR3 and BmOrco. BmOR3 also selectively coupled with Gi proteins, which was dispensable for BmOrco coupling. Moreover, we demonstrated that *trans*-7TM BmOR3 recruited arrestin in an agonist-dependent manner, which indicates an important role for BmOR3–BmOrco complex formation in ionotropic functions. Collectively, our study identified the coupling of G protein and arrestin to a prototype *trans*-7TM PR, BmOR3, and provided important mechanistic insights into the coupling of active PRs to their downstream effectors, including coreceptors, G proteins, and arrestin.

As the most diverse group of animals on earth, insects use invisible chemical signals called pheromones to coordinate their reproductive, aggregation, and alarming behaviors (1, 2, 3). Pheromone molecules are detected by specific receptors expressed in olfactory sensory neurons (OSNs), which are housed in hair-like sensilla at two main chemoreceptive organs, antennae and maxillary palps (4, 5, 6). Pheromone receptor (PR) activation leads to chemoelectrical transduction within receptor-specific OSNs, which sends signals to the same glomeruli in the antennal lobe. The signals are further relayed into higher centers in the brain through secondary projection neurons, where the information is processed and converted to behavior-guiding outputs (7).

Insect PRs belong to the superfamily of olfactory receptors (ORs). These insect ORs have distinct topology compared with human ORs, which have transverse seven transmembrane (*trans*-7TM) bundles *versus cis*-7TM bundles. Whereas common ORs discriminate odorants by a combinatorial receptor code that activates multiple OSNs, PRs are highly specific for pheromones to act through dedicated classes of OSNs (8, 9). Similar to other insect ORs, the activation of PRs couples to their Orco (10). This coupling leads to cation influx, which regulates OSN activities (11, 12). In contrast to PR, which is divergent and confers ligand specificity, Orco is highly conserved across insect species but does not directly bind to pheromones. The deficiency of either PR or its Orco leads to aberrance of pheromone-mediated social behaviors or host selection (8, 13, 14, 15). Most recently, the cryo-EM structure of Orco from the fig wasp *Apocrypta bakeri* was determined, which provided key information regarding Orco homomer assembly and ion pore formation (16). However, the mechanism underlying the functional coupling of pheromone-binding PR and pheromone-free Orco remains largely unknown.

In addition to Orco coupling, several insect PRs have been reported to mediate Gs or Gq activation in heterologous cells upon ligand stimulation (12, 17). Consistently, G protein expression has been identified in insect OSNs. Therefore, certain insect PRs are functionally correlated with G protein-coupled receptors (GPCRs). However, unlike their vertebrate counterparts, which are canonical GPCRs, insect PRs have an inverted topology with an intracellular N terminus and an extracellular C terminus (18, 19). The absence of a conventional mammalian GPCR G protein-binding site questions its capability to couple and activate G proteins (11). In addition, arrestins, which are indispensable regulators of GPCR signaling mediating receptor desensitization, are also functionally involved in the insect olfactory response; however, there is no evidence that insect PRs directly interact with arrestins (20, 21). Therefore, the signaling potential of insect PRs for eliciting traditional G protein or arrestin signaling as that in mammals is highly controversial and awaiting elucidation.

In the present study, using BmOR3 from the silk moth *Bombyx mori* and its coreceptor BmOrco as a template (22), we investigated the structural and functional mechanisms underlying PR–Orco coupling. We designed a series of FlAsH-BRET (fluorescein arsenical helix–bioluminescence resonance energy transfer) sensors to detect the conformational changes of BmRO3 and BmOrco during the coupling process. Combined with the utilization of calcium sensors, we revealed that an agonist-induced conformational change of BmOR3 was transmitted to BmOrco through transmembrane segment 7 (S7) from both receptors, resulting in the activation of BmOrco and cation ion influx. We delineated key interactions, including an ionic lock and a hydrophobic zipper, in stabilizing BmOR3–BmOrco coupling. Further analyses revealed potential Gi protein coupling, which was dependent on a conserved tryptophan (Trp), along with the N terminus–regulated arrestin recruiting capability of BmOR3. Arrestin was found to be critical for the functional coupling of BmOR3 and BmOrco.

## Results

### Bombykal induces BmOR3–BmOrco coupling and conformational changes of BmOrco

**Figure 1:**
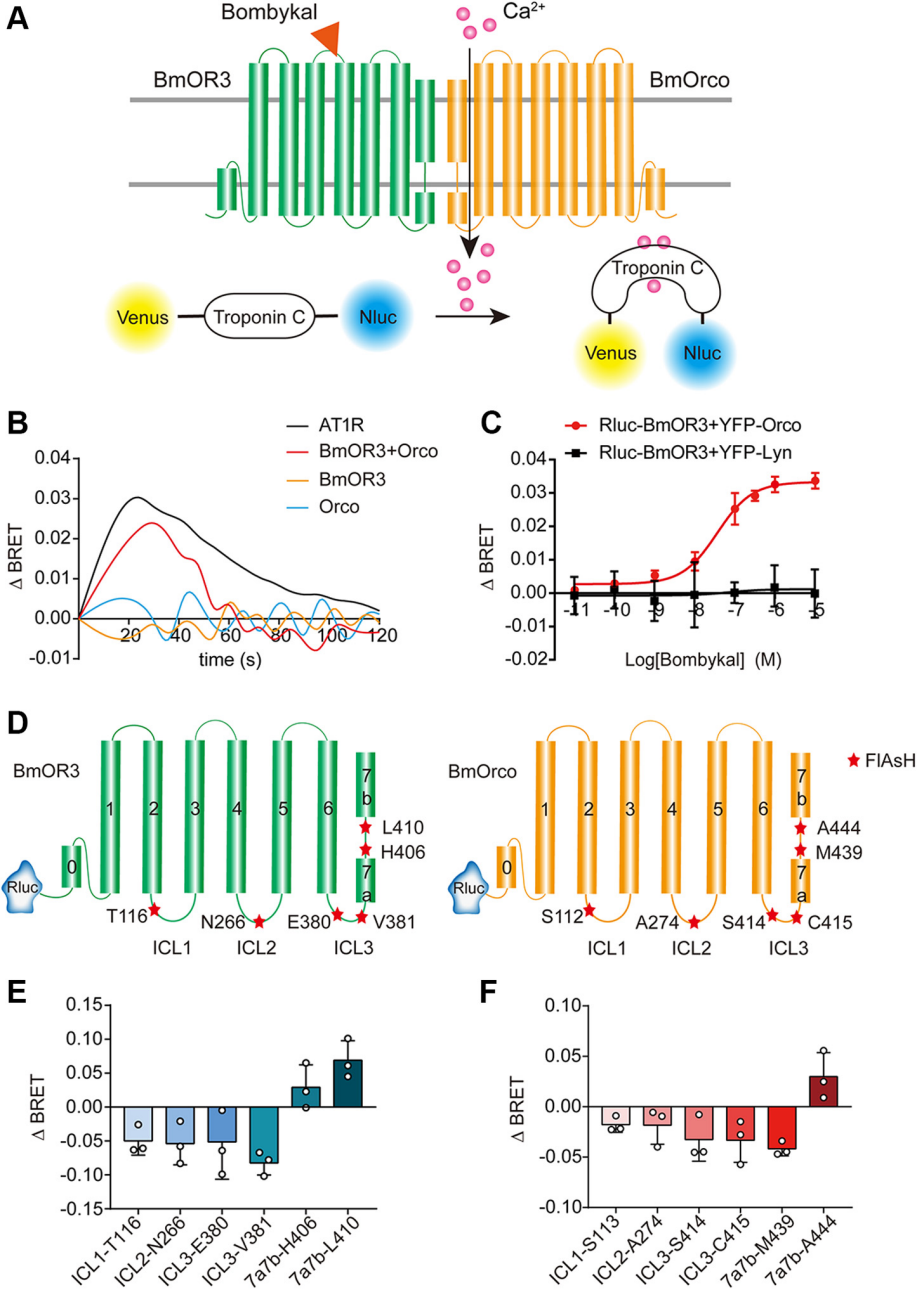
**Bombykal induces BmOR3–BmOrco coupling and conformational changes of BmOrco**. *A*, schematic diagram of the detection of calcium response by using BRET-based calcium sensor CalfluxVTN. *B*, 1 μM bombykal- or AngII-induced calcium influx in CalfluxVTN-expressing HEK293 cells transfected with AT1R, BmOR3, BmOrco, or both BmOR3 and BmOrco. *C*, bombykal-induced dose-dependent coupling of BmOR3 and BmOrco measured by BRET assays. The lyn-YFP was used as the negative control. *D*, schematic diagram of the Rluc-BmOR3-FlAsH and Rluc-BmOrco-FlAsH constructs. *E*, bombykal-induced intramolecular ΔBRET changes in HEK293 cells transfected with the respective Rluc-BmOR3-FlAsH constructs. *F*, bombykal-induced intramolecular ΔBRET changes in HEK293 cells transfected with BmOR3 and the respective Rluc-BmOrco-FlAsH constructs. Data are shown as the mean ± SD of at least three independent experiments. BRET, bioluminescence resonance energy transfer; FlAsH, fluorescein arsenical helix.

It was proposed that divergent insect ORs physically interact with highly conserved Orco to form nonselective cation channels. In the silk moth *B. mori*, BmOrco (also named BmOR2) is coexpressed with BmOR1 or BmOR3 in different olfactory neurons to confer responses to bombykol or bombykal, respectively (17, 22). By using a genetically encoded BRET-based calcium sensor composed of Venus, troponin, and NanoLuc, we investigated the signaling properties of the BmOR3–BmOrco pair by expressing these receptors in heterologous human embryonic kidney 293 (HEK293) cells (23). Calcium binding to troponin leads to a conformational change that brings NanoLuc closer to Venus and results in resonance energy transference (Fig. 1*A*). We found that the coexpression of BmOR3 and BmOrco in HEK293 cells, but not the expression of BmOR3 or BmOrco alone, elicited robust calcium influx in response to stimulation with the specific BmOR3 agonist bombykal (Fig. 1*B*). The bombykal-stimulated Ca^2+^ response through BmOR3/BmOrco was approximately 70% of that induced by angiotensin II through activating AT1R, when the receptors were expressed at similar levels (Figs. 1*B* and S1, *A* and *B*). To verify the physical interaction between BmOR3 and BmOrco, we used a BRET assay by tagging Renilla luciferase (Rluc) to the N terminus of BmOR3 and YFP to BmOrco. Intriguingly, a specific saturation BRET signal was detected between Rluc-BmOR3 and YFP-BmOrco in a plasmid concentration–dependent manner, but not between Rluc-BmOR3 and the plasma membrane–anchored Lyn-YFP (YFP fused to the fatty acylation motif of Lyn-kinase), which were used as the negative control (24), indicating a constitutive interaction between BmOR3 and BmOrco. The BRET50 for the BmOR3–BmOrco heterodimer occurred at about a 1:1 receptor expression ratio (Fig. S1, *C* and *D*). Moreover, the bombykal elicited a concentration-dependent BRET signal increase between Rluc-BmOR3 and YFP-BmOrco (EC_50_: 36.04 ± 3.89 nM) but not between Rluc-BmOrco and the plasma membrane marker Lyn-YFP (Fig. 1*C*). The above results indicated a functional coupling and physical interaction between BmOR3 and its coreceptor BmOrco, which was augmented in response to bombykal stimulation.

To delineate the landscape of the bombykal-induced conformational changes of BmOR3 and BmOrco, we generated a panel of conformational BRET sensors by incorporating Rluc at the N terminus of BmOR3 or BmOrco and FlAsH probes (CCPGCC) into specific positions of intracellular loops (ICLs) or loops within S7 (loop^7a-7b^) in both receptors (Fig. 1*D*). Such FlAsH-BRET sensors have been successfully used to study the conformational changes of receptors and the interactions between the ligands and receptors (25, 26, 27, 28). We first controlled the equal expression levels of different BmOR3 or BmOrco constructs on the cell surface by the ELISA (Fig. S1, *E* and *F*). Bombykal induced significant decreases in the intramolecular BRET signal at ICL1, ICL2, and ICL3 of BmOR3, suggesting that these cytoplasmic parts moved away from the N terminus in response to bombykal stimulation (Fig. 1*E*). In contrast, the BRET signal between the N-terminal Rluc and the FlAsH labeling at loop^7a-7b^ increased, suggesting that the C-terminal helical kink moved close to the N terminus of BmOR3 in response to bombykal stimulation (Fig. 1*E*).

Intriguingly, bombykal induced the conformational rearrangement of BmOrco with a different signature than BmOR3. This difference was reflected in the lower part of loop^7a-7b^ (position H406 in BmOR3 and position M439 in BmOrco), which moved away from the N terminus in BmOrco but remained close in BmOR3 (Fig. 1, *E* and *F*). This discrepancy suggested that a significant distortion of loop^7a-7b^ in BmOrco takes place in response to bombykal stimulation. The movement away of the ICLs from the N terminus in both BmOR3 and BmOrco, along with the distortion of the loop^7a-7b^ in BmOrco, may serve as key conformational rearrangement features for the coupling between BmoR3 and BmOrco and BmOrco-mediated cation influx.

### Features of conformational changes during BmOR3–BmOrco coupling

**Figure 2:**
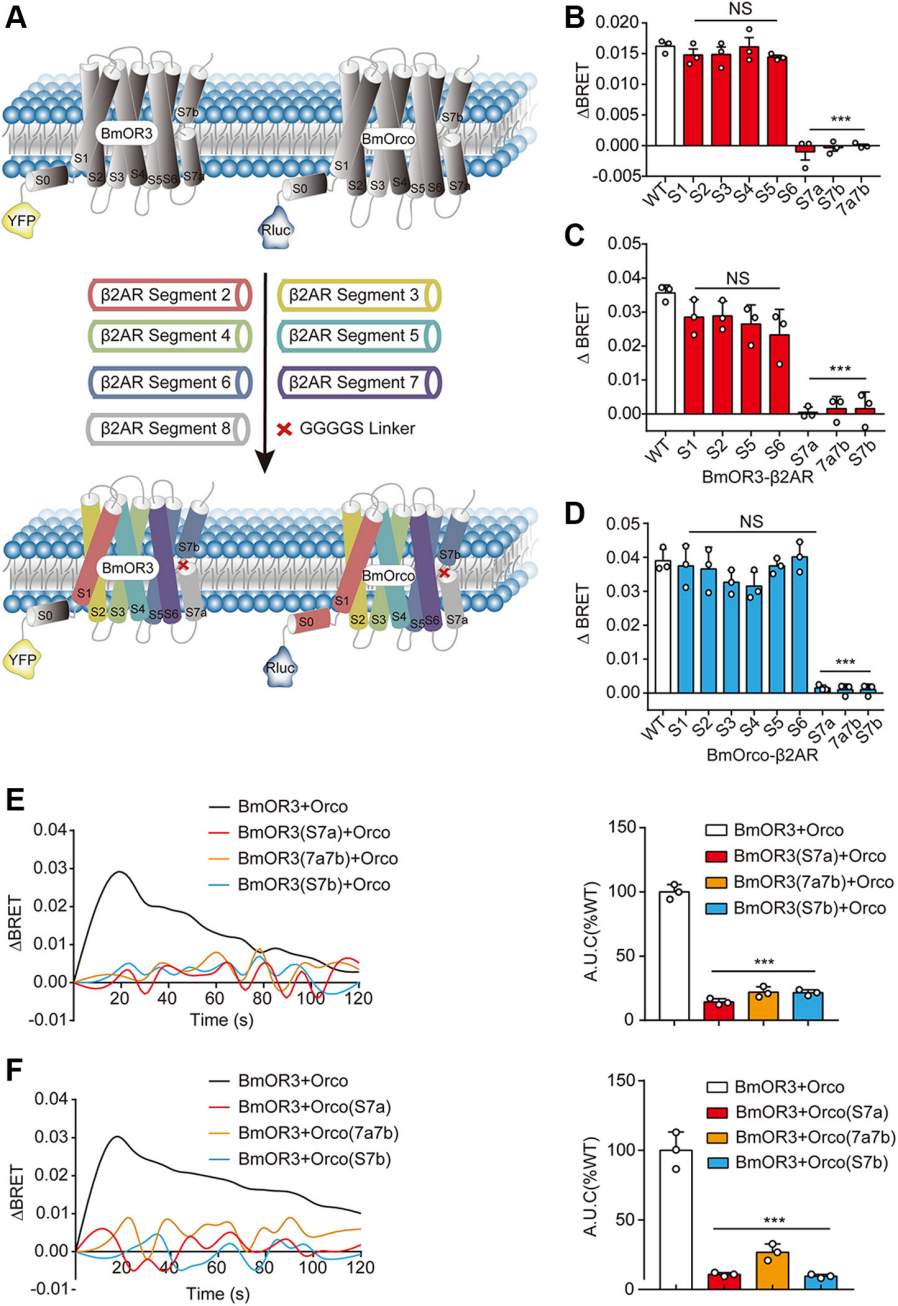
**S7 segments of BmOR3 and BmOrco mediate the functional coupling**. *A*, schematic diagram showing the replacement of transmembrane segments and loops of BmOR3 or BmOrco by corresponding elements from β2AR or linker GGGGS, respectively. *B*, bombykal-induced intramolecular ΔBRET changes in HEK293 cells transfected with Rluc-BmOrco-FlAsH (position M439) together with WT or chimeric BmOR3. *C*, bombykal-induced intermolecular ΔBRET changes in HEK293 cells transfected with Rluc-BmOrco and WT or chimeric YFP-BmOR3. *D*, bombykal-induced intermolecular ΔBRET changes in HEK293 cells transfected with YFP-BmOR3 and WT or chimeric Rluc-BmOrco. *E*, *left panel*, bombykal-induced calcium influx in CalfluxVTN-expressing HEK293 cells transfected with BmOrco and WT or chimeric BmOR3; *right panel*, quantification of the area under the curve (AUC) of calcium influx. Data were normalized to the calcium response in cells transfected with WT BmOR3 and BmOrco. *F*, *left panel*, bombykal-induced calcium influx in CalfluxVTN-expressing HEK293 cells transfected with BmOR3 and WT or chimeric BmOrco; *right panel*, quantification of the area under the curve (AUC) of calcium influx. Data were normalized to the calcium response in cells transfected with WT BmOR3 and BmOrco. *B–F*, cells transfected with chimeric BmOR3 or BmOrco were compared with cells transfected with WT receptors. ∗∗∗*p* < 0.001. Data are shown as the mean ± SD of at least three independent experiments. Data statistics were analyzed using one-way ANOVA with Dunnett's post hoc test. BRET, bioluminescence resonance energy transfer; FlAsH, fluorescein arsenical helix; NS, no significant difference; S7, segment 7.

The cryo-EM structure of *A. bakeri* Orco revealed that the channel pore mainly consists of helix S7b, whereas the whole homotetramer is stabilized by a cytosolic anchor domain and segments from S4, S5, S6, and S7a (16). However, the structural basis of functional coupling between PR and Orco remains unclear. To identify the key components that mediate the interaction between BmOR3 and BmOrco, we replaced the individual transmembrane segment of BmOR3 or BmOrco with the corresponding helix from a prototype GPCR, β2 adrenoreceptor (β2AR), and we studied the effects of these substitutions on bombykal-induced BmOR3–BmOrco conformational changes (Fig. 2*A*). We assumed that each helix bundle of the 7TM receptors could be regarded as an independent structural element in this substitution study. The equal cell surface expression levels of different BmOR3 or BmOrco chimeras were verified (Fig. S2, *A* and *B*). Notably, the substitution of S3 or S4 in BmOR3 caused the deficiency of receptor expression, and these two chimeras were excluded from further functional studies. The S7 segments of both BmOR3 and BmOrco were found to be critical for BmOR3–BmOrco coupling because the substitution of any element within S7, including S7a, S7b, and loop^7a-7b^, abolished the bombykal-induced intramolecular BRET signals between the N-terminal Rluc and the FlAsH inserted at loop^7a-7b^ (position M439) in BmOrco (Fig. 2*B*).

To further clarify the mechanism underlying S7 replacement–induced BmOrco inactivation, we inspected the effects of segment substitution on BmOR3–BmOrco coupling using an intermolecular BRET assay, which directly measures the energy transference from Rluc-tagged BmOrco to YFP-tagged BmOR3. Whereas the substitution of the other segments showed negligible effects on bombykal-induced BmOR3–BmOrco coupling, the substitution of S7 elements in either BmOR3 or BmOrco by the β2AR counterpart led to complete collapse of the complex formation (Fig. 2, *C* and *D*). Accordingly, substitution of the S7 element in either BmOR3 or BmOrco dramatically decreased the bombykal-induced calcium influx, further confirming the indispensable role of S7 in BmOR3–BmOrco for ionotropic functions (Fig. 2, *E* and *F*). Collectively, these data demonstrated that the functional coupling of BmOR3 to BmOrco is mediated by the S7 segments of both receptors.

### Key structural motifs mediating BmOR3–BmOrco coupling

**Figure 3:**
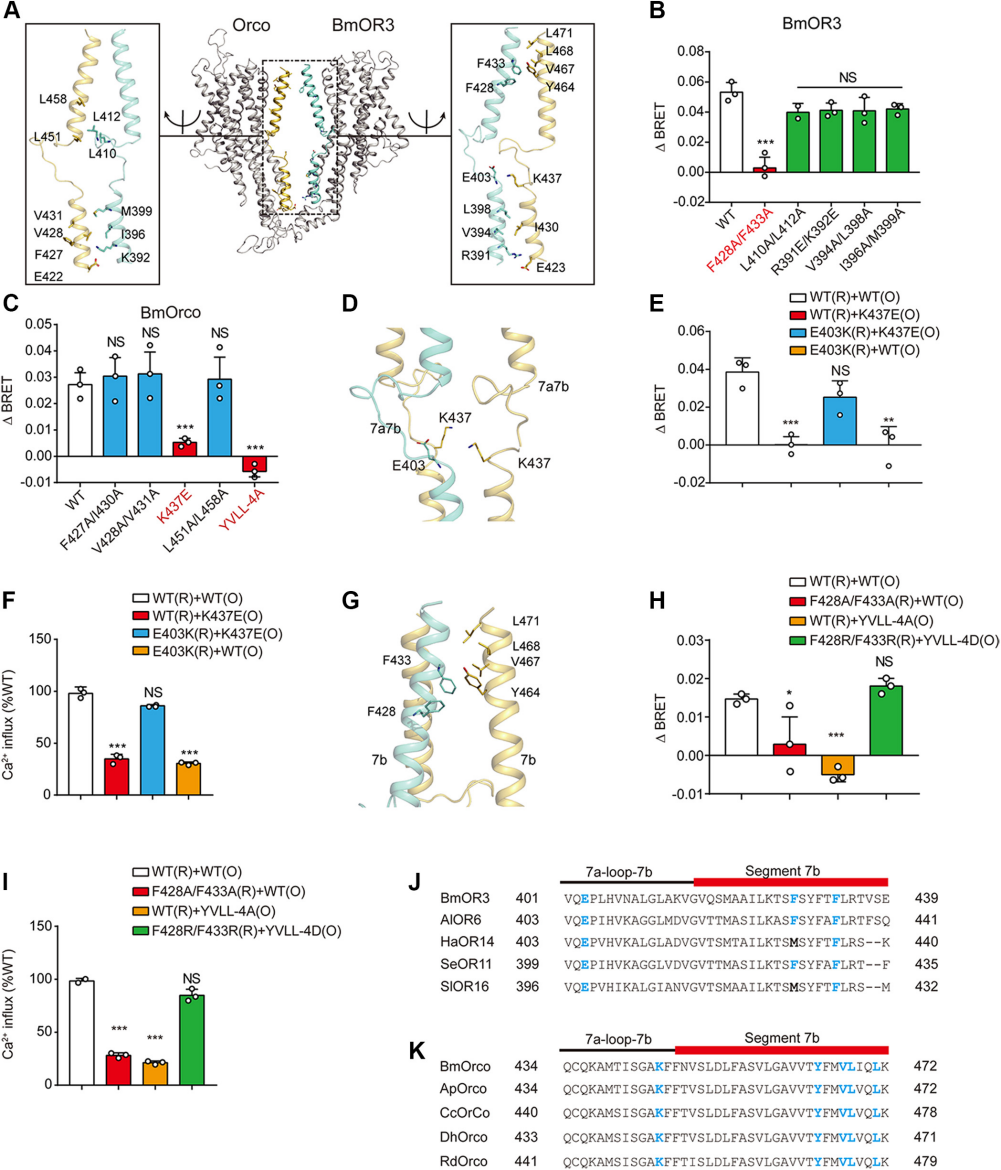
**Key motifs in S7 govern the functional coupling between BmOR3 and BmOrco**. *A*, schematic representation showing the potential interacting residues in the S7 segments of BmOR3 (*cyan*) and BmOrco (*yellow*). *B*, bombykal-induced intermolecular ΔBRET changes in HEK293 cells transfected with Rluc-BmOrco and WT or mutated YFP-BmOR3. *C*, bombykal-induced intermolecular ΔBRET changes in HEK293 cells transfected with YFP-BmOR3 and WT or mutated Rluc-BmOrco. *D*, potential interaction between BmOrco-K437 and BmOR3-E403. *E*, effects of mutations of BmOrco-K437 or BmOR3-E403 on bombykal-induced BmOR3–BmOrco coupling. *F*, effects of mutations of BmOrco-K437 or BmOR3-E403 on bombykal-induced calcium response. *G*, potential interactions between the hydrophobic residues at S7 segments of BmOR3 and BmOrco. *H*, effects of mutations of hydrophobic residues at S7 segment of BmOrco or BmOR3 on bombykal-induced BmOR3–BmOrco coupling. *I*, effects of mutations of hydrophobic residues at S7 segment of BmOrco or BmOR3 on bombykal-induced calcium response. *J*, sequence alignments of BmOR3 S7 with ORs from different insect species with key residues highlighted. *K*, sequence alignments of BmOrco S7 with Orcos from different insect species with key residues highlighted. *B–I*, cells transfected with BmOR3 or BmOrco mutants were compared with cells transfected with WT receptors. ∗*p* < 0.05; ∗∗*p* < 0.01; ∗∗∗*p* < 0.001. Data are shown as the mean ± SD of at least three independent experiments. Data statistics were analyzed using one-way ANOVA with Dunnett's post hoc test. “R” in the brackets represents BmOR3, and “O” in the brackets represents BmOrco. BRET, bioluminescence resonance energy transfer; NS, no significant difference; OR, olfactory receptor; S7, segment 7.

To further delineate the structural mechanism underlying the coupling between BmOR3 and BmOrco, we built a simulated BmOR3–BmOrco interaction model in SWISS-MODEL using the cryo-EM structure of the Orco homomer as a template (16). Based on this model, a series of hydrophobic clusters and charged residues located in the S7 segments of both BmOR3 and BmOrco were identified as potential interacting sites of these two receptors (Fig. 3*A*). We then performed charge-reversed or alanine mutagenesis scanning to delineate the molecular basis of BmOR3–BmOrco coupling. Notably, mutation of the hydrophobic pair F428/F433 to Ala in BmOR3 severely impaired the bombykal-induced BRET signal between BmOR3 and BmOrco, whereas mutation of L410A/L412A, R391A/K392E, V394A/L398A, or I396A/M399A had no significant effects (Fig. 3*B*). The reversion of the charge property of K437 in BmOrco by E substitution or mutation of the hydrophobic zipper Y464/V467/L468/L471 to Ala also abolished the bombykal-induced association of BmOR3 and BmOrco. However, other substitutions, such as F427A/I430A, V428A/V431A, and L451A/L458A, showed no significant effects (Fig. 3*C*). Notably, all the above alanine mutations did not significantly affect the cell surface expression levels of BmOR3 or BmOrco (Fig. S2, *C* and *D*). Therefore, the F248/F433 pair, the hydrophobic zipper Y464/V467/L468/L471, and the positively charged K437 are the key residues in mediating the bombykal-elicited interaction of BmOR3 and BmOrco.

In our simulated model, K437 in loop^7a-7b^ of BmOrco was in close proximity to glutamate (E403) in loop^7a-7b^ of BmOR3 (Fig. 3*D*). We suspected that BmOR3-E403 and BmOrco-K437 constitute a charged pair that is critical for the interaction between BmOR3 and BmOrco. Consistently, the mutation of E403 to K in BmoR3 led to significant decreases in both the intermolecular BRET signal and calcium influx, similar to those caused by the K437E mutation of BmOrco. However, the double mutation of BmOR3–E403K and BmOrco–K437E restored the BRET signal and calcium signaling (Fig. 3, *E* and *F*). Therefore, the charged residue pair of BmOR3-E403–BmOrco-K437 represents an important “ionic lock” in regard to mediating BmOR3–BmOrco coupling. Moreover, the hydrophobic patches F428/F433 of BmOR3 and Y464/V467/L468/L471 of BmOrco are spatially close to each other, suggesting that they might form hydrophobic interactions (Fig. 3*G*). Consistently, the alanine mutation of each of the abovementioned elements caused significant impairment of BmOR3–BmOrco complex formation and decreased calcium influx in response to agonist stimulation. In contrast, simultaneous mutations of the BmOR3–F428/433 cluster to positively charged Arg and the BmOrco–Y464/V467/L468/L471 cluster to negatively charged Asp restored the intermolecular BRET signal and calcium signaling (Fig. 3, *H* and *I*). Therefore, these results suggested that a hydrophobic zipper is formed between BmOR3 and BmOrco during their functional coupling. Importantly, the newly defined ionic lock and hydrophobic zipper are highly conserved across insect OR systems, which suggests a potential common mechanism underlying the coupling of insect OR-Orco (Fig. 3, *J* and *K*).

### Coupling between BmOR3 and Gi

**Figure 4:**
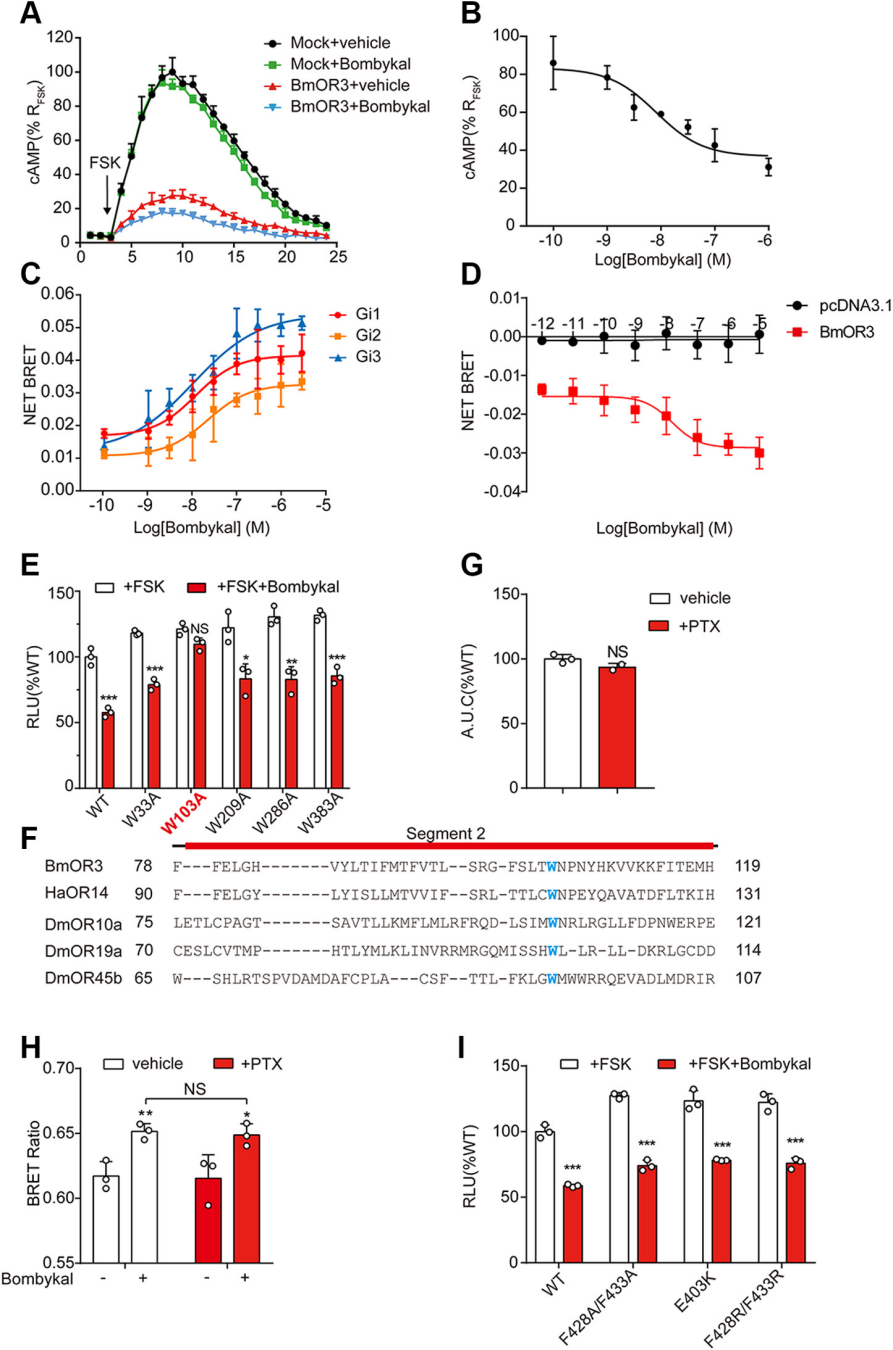
**BmOR3 couples to Gi in a conserved tryptophan-dependent manner**. *A*, real-time measurement of forskolin-induced cAMP production in BmOR3- or pcDNA-transfected GloSensor-expressing HEK293 cells upon vehicle or bombykal stimulation. *B*, bombykal-induced dose-dependent decrease in forskolin-stimulated cAMP production in GloSensor-expressing HEK293 cells transfected with BmOR3. Data were normalized to the forskolin–stimulated maximal cAMP response in BmOR3-expressing HEK293 cells. *C*, bombykal-induced dose-dependent coupling of BmOR3 to Gαi measured by G protein recruitment BRET assay. The net BRET was determined by subtracting the BRET values in cells transfected only with Gαi-Rluc. *D*, bombykal-induced dose-dependent Gi activation measure by G protein dissociation BRET assay in HEK293 cells transfected with Gi BRET probes together with BmOR3 or with pcDNA3.1. *E*, effect of tryptophan mutations of BmOR3 on bombykal-induced cAMP reduction in GloSensor-expressing HEK293 cells. *F*, sequence alignments of BmOR3 S2 with ORs from different insect species with conserved tryptophan highlighted. *G*, bombykal-induced calcium response in BmOR3 and BmOrco transfected HEK293 cells pretreated with vehicle or 100 ng/ml pertussis toxin (PTX). *H*, bombykal-induced intermolecular BRET between BmOR3-YFP and Rluc-BmOrco in HEK293 cells pretreated with vehicle or PTX. *I*, effects of mutations of resides governing BmOR3–BmOrco coupling on the bombykal-induced cAMP reduction in GloSensor-expressing HEK293 cells. *E*, *I*, cells treated with forskolin and bombykal were compared with cells treated with forskolin only. ∗*p* < 0.05, ∗∗*p* < 0.01, ∗∗∗*p* < 0.001. *G* and *H*, cells treated with bombykal were compared with cells treated with vehicle. ∗*p* < 0.05, ∗∗*p* < 0.01. Cells treated with PTX were compared with cell treated with vehicle. Data are shown as the mean ± SD of at least three independent experiments. Data statistics were analyzed using one-way ANOVA with Dunnett's post hoc test. BRET, bioluminescence resonance energy transfer; NS, no significant difference; ORs, olfactory receptors.

The G protein–coupling potential of insect PRs is controversial, most likely because they have a distinct transmembrane topology compared with classic GPCRs in mammals. To characterize the G protein signaling properties of BmOR3, we expressed the receptors in HEK293 cells. The cAMP signaling was investigated because the insect olfactory functionality has been reported to be affected by mutations disturbing the cAMP transduction pathway (29). Unexpectedly, overexpression of BmOR3 led to a 60% reduction in forskolin-induced intracellular cAMP production, which indicates potential constitutive Gi activity (Fig. 4*A*). Moreover, bombykal stimulated a concentration-dependent decrease in forskolin-induced cAMP in BmOR3-transfected cells but not in mock cells (Fig. 4*B*). To further verify the Gi-coupling property of BmOR3, we used a BRET assay by monitoring the direct interaction between YFP-BmOR3 and Rluc-labeled different Gi protein subtypes. This method has been used for detecting the coupling of CXCR4 with Gi proteins (30). Our results showed that bombykal induced dose-dependent coupling of Rluc-tagged Gi proteins to YFP-fused BmOR3 (EC_50_: 11.69 ± 1.19 nM, 21.98 ± 3.22 nM, 10.56 ± 2.06 nM) for Gi1, Gi2, and Gi3 (Fig. 4*C*). Notably, the observed net BRET value in the absence of bombykal stimulation supported the constitutive coupling of BmOR3 to Gi protein. To further dissect the G protein signaling properties of BmOR3, we used G protein dissociation BRET assay, which measures the BRET between GFP10-tagged Gγ and Rluc8-inserted Gα subunits (31). Consistent with the results of G protein recruitment assay, the bombykal induced dose-dependent Gi activation through BmOR3 as revealed by the decrease of the BRET signal (EC_50_: 16.45 ± 5.04 nM) (Fig. 4*D*). In contrast, the bombykal stimulation did not induce Gs, Gq, Go, or Gz activation (Fig. S3, *A*–*D*). Therefore, these results collectively provided direct evidence that BmOR3 is a Gi protein–coupled receptor.

In many mammalian GPCRs, a Trp is located within a highly conserved CWxP motif in TM6, which plays an important role in mediating the transition of the receptor from inactive to active conformations (32). The conformational change of this Trp has been observed in the activation process of a variety of GPCRs, such as rhodopsin, cannabinoid receptor 1, and A_2A_ adenosine receptor (33, 34). We next sought to explore whether a large hydrophobic Trp also participates in the activation processes of insect PRs, although they have transmembrane topology that is distinct from that of canonical GPCRs. We mutated five tryptophan residues, which are located at different transmembrane segments, to Ala and then tested the mutagenesis effects on Gi signaling. Interestingly, only W103A completely abolished the bombykal-induced cAMP reduction in BmOR3-overexpressing cells (Fig. 4*E*). Furthermore, sequence alignment revealed the conservation of this tryptophan in S2 in several insect species. Therefore, tryptophan might exist in several insect PRs to participate in the activation process of insect PRs to induce G protein signaling (Fig. 4*F*). Intriguingly, pretreatment with pertussis toxin (PTX), a specific Gi inhibitor, showed no significant effects on bombykal-induced BmOR3–BmOrco complex formation or complex-regulated calcium influx, suggesting that Gi coupling and BmOrco coupling are two independent processes (Fig. 4, *G* and *H*). Consistently, mutations of the ionic lock or hydrophobic zipper, which were shown to induce the collapse of the BmOR complex, had no significant effects on Gi-cAMP signaling (Fig. 4*I*). Furthermore, the bombykal-induced BmOR3–Gi interactions were not significantly affected by BmOrco incorporation (Fig. S3, *E* and *F*).

### Arrestin couples to BmOR3 and mediates BmOR3 functions

**Figure 5:**
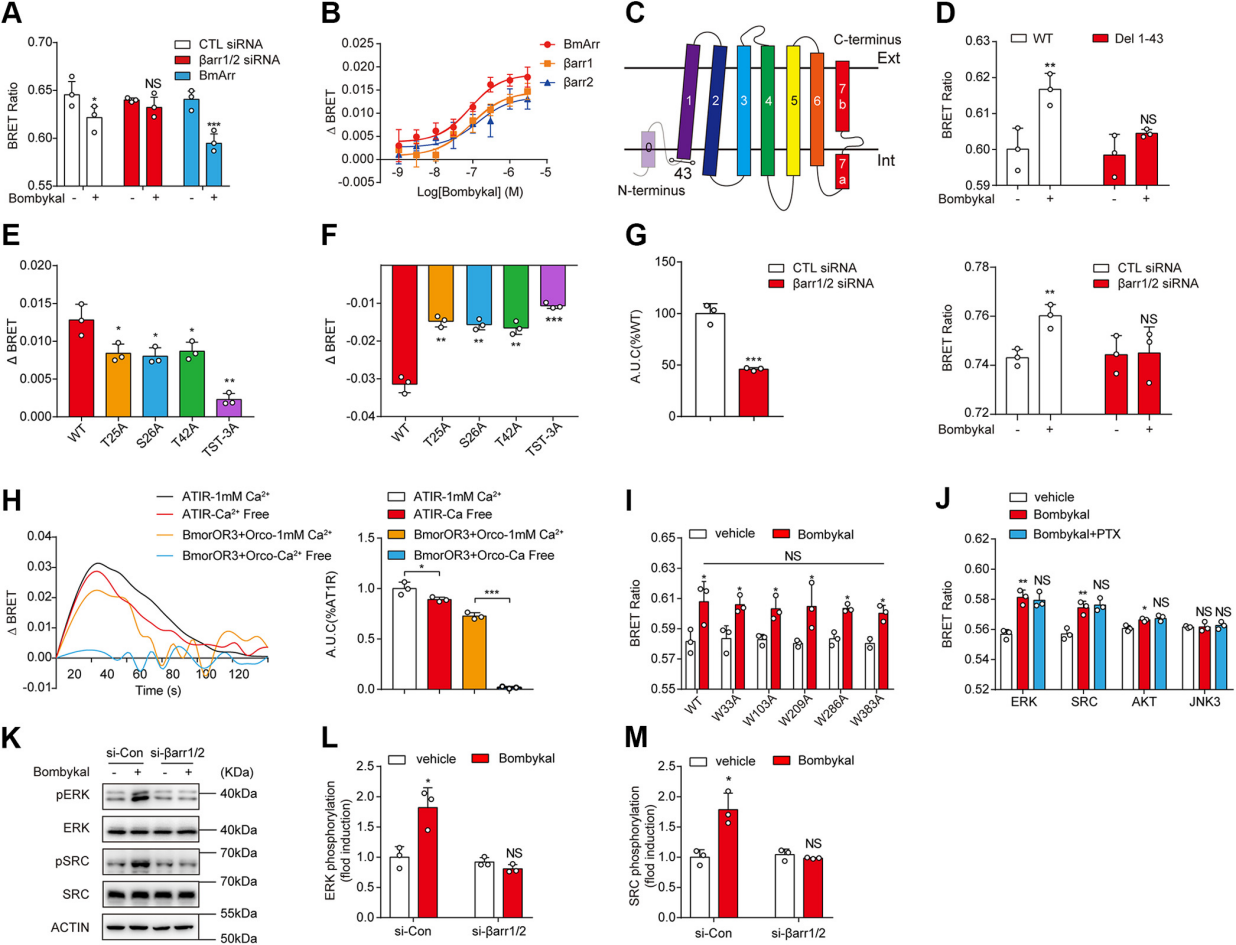
**Arrestin mediates BmOR3 internalization and activities**. *A*, bombykal-induced BmOR3 internalization detected by a BRET-based trafficking sensor in HEK293 cells transfected with control siRNA, βarr1/2 siRNA, or BmArr. *B*, bombykal-induced dose-dependent recruitment of βarr1, βarr2, or BmArr to BmOR3 measured by BRET assay. *C*, schematic diagram of truncated BmOR3 with the first 43 residues deleted. *D*, bombykal-induced recruitment of BmArr to BmOR3 in HEK293 cells transfected with Rluc-BmArr and YFP-WT-BmOR3 or YFP-(Del 1–43)-BmOR3. *E*, effects of mutations of potential phosphorylation sites at the N terminus of BmOR3 on bombykal-stimulated BmArr recruitment. *F*, effects of mutations of potential phosphorylation sites at the N terminus of BmOR3 on bombykal-stimulated BmOR3 internalization. *G*, *left panel*, bombykal-induced calcium responses in BmOR3 and BmOrco expressing HEK293 cells transfected with control siRNA or βarr1/2 siRNA. *Right panel*, bombykal-induced intermolecular BRET between BmOR3-YFP and Rluc-BmOrco in HEK293 cells transfected with control siRNA or βarr1/2 siRNA. *H*, representative curves (*left*) and quantification (*right*) of 1 μM bombykal or AngII-induced calcium responses in BmOR3 or AT1R-overexpressing HEK293 cells incubated with Ca-containing or Ca-free buffer. *I*, bombykal-induced recruitment of BmArr to BmOR3 in HEK293 cells transfected with Rluc-BmArr and WT or mutant YFP-BmOR3. *J*, bombykal-induced interaction of BmArr-Rluc with YFP-tagged ERK, SRC, AKT, or JNK3 in HEK293 cells transfected with BmOR3 and pretreated without or with PTX. *K–M*, representative blots and quantification of bombykal-stimulated ERK or SRC phosphorylation in BmOR3-expressing HEK293 cells transfected with control siRNA or βarr1/2 siRNA. *A*, *D*, *I–M*, cells treated with bombykal were compared with cells treated with vehicle. ∗*p* < 0.05; ∗∗*p* < 0.01; ∗∗∗*p* < 0.001. *E* and *F*, cells transfected with BmOR3 mutants were compared with cells transfected with WT BmOR3. ∗*p* < 0.05; ∗∗*p* < 0.01; ∗∗∗*p* < 0.001. *G*, *left*, ∗∗∗*p* < 0.001. Cells transfected with βarr1/2 siRNA were compared with cells transfected with control siRNA. ∗∗*p* < 0.01. Cells treated with bombykal were compared with cells treated with vehicle. ns, no significant difference. *H*, cells incubated in Ca-free buffer were compared with cells incubated with Ca-containing buffer. ∗*p* < 0.05; ∗∗∗*p* < 0.001. *J*, cells treated with PTX were compared with cells treated with control vehicle. Data are shown as the mean ± SD of at least three independent experiments. Data statistics were analyzed using one-way ANOVA with Dunnett's post hoc test. BRET, bioluminescence resonance energy transfer; NS, no significant difference.

In mammals, activated GPCRs are commonly phosphorylated by G-protein coupled receptor kinases, which promote the recruitment of β-arrestins. Arrestins mediate receptor desensitization and internalization, preventing receptor overactivation (35). Arrestins are also present in most insect cells. Although arrestins mediate the desensitization of *cis*-7TM receptors, whether arrestins regulate *trans*-7TM insect PRs has not been characterized. We examined whether arrestin participates in BmOR3 internalization using BRET-based trafficking sensors. The Lyn-YFP was utilized as the plasma membrane marker to quantify the agonist-induced internalization of BmOR3. A similar approach has been used in our previous studies (36). Bombykal stimulation caused a significant decrease in the BRET signal, which indicates the internalization of BmOR3 (Fig. 5*A*). Whereas pretreatment with β-arrestin1/2 siRNA completely abolished bombykal-induced BmOR3 internalization, the overexpression of *B. mori* intrinsic arrestin (BmArr1) facilitated BmOR3 trafficking (Fig. 5*A*). We then used an intermolecular BRET assay to investigate the recruitment of arrestins by BmOR3. Bombykal robustly stimulated the recruitment of β-arrestin-1, β-arrestin-2, and BmArr1 to BmOR3 in HEK293 cells in a concentration-dependent manner (91.08 ± 1.61 nM, 150.87 ± 21.33 nM, 90.03 ± 11.06 nM, respectively) (Fig. 5*B*).

Collectively, the above results indicated that bombykal induced the recruitment of arrestin to BmOR3, which in turn regulated BmOR3 internalization. It is known that the phosphorylated C termini or ICL3 of mammalian GPCRs are major docking places for β-arrestin recruitment (37, 38, 39). However, insect PRs have an inverted membrane topology compared with mammalian GPCRs. Therefore, we hypothesized that BmOR3 interacted with arrestin *via* the intracellular N terminus and accordingly created a truncated BmOR3 with the first 43 amino acids deleted (Fig. 5*C*). Although the expression levels on the cell surface remained normal, truncated BmOR3 showed an impaired response of arrestin recruitment in response to bombykal stimulation compared with the WT receptor, which suggests the functional importance of the N terminus of insect PR in arrestin engagement (Figs. 5*D* and S4*A*). There are two threonine (T25 and T42) and one serine (S26) at the N terminus of BmOR3. To explore the functional roles of these potential phosphorylation sites, we performed alanine substitutions at these sites and found that mutation of either of these three residues led to decreased arrestin recruitment and receptor internalization in response to bombykal stimulation (Fig. 5, *E* and *F*). Notably, the mutants showed similar expression levels as the WT receptor, thus excluding the possibility that the observed BRET effects were caused by different receptor expression (Fig. S4*A*). Collectively, the results suggested an indispensable role of these potential phosphorylation sites at the N terminus of BmOR3 in arrestin recruitment and receptor internalization.

In parallel to G protein signaling, recent studies in mammalian GPCRs have revealed that arrestins redirect signaling to numerous G protein-independent pathways in addition to their desensitization function (36, 37, 38, 40, 41, 42). Therefore, we examined whether arrestin had potential independent functions after PR activation. Indeed, the knockdown of β-arrestins significantly reduced bombykal-induced calcium influx through BmOR3–BmOrco, which was accompanied by the collapse of the receptor complex, suggesting an important regulatory role of arrestin protein in BmOR3 functions (Fig. 5*G*). (The knockdown efficiency of β-arrestin-1 and β-arrestin-2 was determined as 70% and 60%, respectively (Fig. S4, *B* and *C*).) To further dissect the mechanism underlying arrestin-mediated Ca^2+^ response downstream of BmOR3, we examined the contribution of extracellular Ca^2+^ influx in the [Ca^2+^]_i_ increase. Notably, whereas the Gq-coupled AT1R-induced Ca^2+^ signal was reduced by approximately ∼10% in the Ca^2+^-free incubating buffer, the bombykal-stimulated Ca^2+^ signal through BmOR3–BmOrco was completely abolished, indicating that the calcium response downstream of BmOR3–BmOrco was mediated by extracellular Ca^2+^ influx rather than intracellular Ca^2+^ release (Fig. 5*H*). Therefore, the β-arrestins mediate Ca^2+^ response mainly by regulating the structural and functional integrity of the BmOR3–BmOrco complex.

Furthermore, we found that BmOR3-arrestin interaction is relatively independent of the G protein because bombykal-induced β-arrestin recruitment to BmOR3 was not affected by PTX pretreatment or tryptophan mutations, including the large hydrophobic residue of W103 (Fig. 5*I* and S4*D*). In contrast, the BmOR3–arrestin interaction was enhanced by BmOrco incorporation in a dose-dependent manner, suggesting the formation of a functional ternary complex (Fig. S4, *E* and *F*). Moreover, by using intermolecular BRET assay, we demonstrated that bombykal-induced BmOR3 activation led to increased association of β-arrestin with multiple downstream signaling molecules, including ERK, SRC, and AKT, which was not significantly affected by PTX pretreatment (Fig. 5*J*). The functionality of these YFP-tagged signaling acceptors, including ERK, SRC, AKT, and JNK, have been validated by BRET assay performed in HEK293 cells overexpressing AT1R, which has been shown to activate these downstream kinases through β-arrestin (43) (Fig. S4*G*). Importantly, the phosphorylation levels of ERK and SRC were significantly elevated upon bombykal stimulation, which were abrogated in β-arrestin siRNA-transfected cells (Fig. 5, *K*–*M*). These results confirmed the β-arrestin-mediated signaling downstream of BmOR3.

Collectively, we not only provided evidence that BmOR3 directly recruits arrestin but also revealed an unprecedented functional role of arrestin in BmOR3 activity regulation.

## Discussion

### Agonist-induced conformational transmission underlies the coupling between PR and Orco

Insects have evolved sophisticated olfactory systems with high sensitivity to detect and interpret odorant and pheromone information in the environment (2, 7). In contrast to mammals and nematodes, insects possess a unique class of PRs that have an inverted membrane topology and couple to a highly conserved Orco (16, 44, 45). Whereas only the PR binds to ligands, the functional heterocomplex formed by the PR and Orco regulates ionotropic channel functions. However, it remains unclear how the complex is formed and functionally coupled. Herein, we demonstrated that agonist-induced conformational changes in BmOR3 were transmitted to BmOrco through direct engagement between these two membrane proteins. An ionic lock and a hydrophobic network composed of residues from S7 of both receptors were found to be essential for complex formation. The key residues involved in these interactions are highly conserved across different insect species, which suggests a common mechanism underlying PR-Orco conformational signaling. For example, Y478 in *Drosophila melanogaster* Orco, which is the equivalent residue of Y464 in BmOrco, was shown to be a critical component of the ion pore. *D. melanogaster* Orco harboring the alanine mutation of Y478 failed to form functional complexes with multiple ORs, such as Or59b, Or85a, Or22a, and Or85b, highlighting the important role of this key residue (46, 47).

FlAsH-BRET sensors enabled us to reveal the conformational transition from BmOR3 to BmOrco in a spatial manner. The differential distortion of loop^7a-7b^ in BmOR3 and BmOrco might suggest an important role of this segment in fine-turning complex formation and ion channel functions. Therefore, techniques with a high temporary resolution, such as single-molecule FRET, would be helpful in the future for comprehensive delineation of PR-Orco conformational signaling.

### Coupling of an insect PR to G protein

Although G protein signaling elements such as cAMP and PKC are functionally involved in insect olfactory signal transduction, the direct coupling of insect PRs with G proteins has been controversial, especially considering that most of the consensus sequences for G protein binding in canonical GPCRs are absent in the cytoplasmic parts of insect PRs (48, 49). For instance, whereas BmOR1, another male-specific PR in *B. mori*, was suspected to activate the Gq pathway, studies using genetic and pharmacological interference have revealed that *Drosophila* ORs signal independently of G proteins (11, 17, 19, 50). In the present study, we provided direct evidence that BmOR3 functionally couples to Gi protein, which might be applicable to at least a subset of insect ORs. Intriguingly, the Gi signaling of BmOR3 is dispensable for bombykal-induced BmOR3–BmOrco complex formation and ionotropic functions. Similar results have also been reported for BmOR1 because treatment with G protein inhibitors had no effect on the agonist-stimulated ionic current response of BmOR1–BmOrco (11). Therefore, the functional output of G protein signaling downstream of BmORs remains to be clarified. Considering the divergent expression of G protein subtypes, such as Gs, Gq, Gi, and Go, in insect OSNs (51, 52), it is reasonable that insect PRs might initiate different signaling pathways by coupling to different G proteins, similar to mammalian GPCRs. Therefore, the functional profiling of G protein pathways is needed to thoroughly delineate the signaling properties of target insect PRs. Moreover, in addition to its roles in pheromone detection, the insect PR system regulates other important physiological functions. For example, the loss of PR function in ants dramatically impairs the morphology of OSNs and the antennal lobe to which OSNs project, which suggests a crucial role of the PR system in neuroanatomical plasticity (14, 15). Therefore, the functional significance of G protein–mediated pathways downstream of insect PRs, especially their potential participation in neural development, deserves further exploration.

### Coupling of an insect PR to arrestin

Arrestin functions as a signal terminator by mediating GPCR desensitization and internalization. Several different arrestin isoforms have been identified in insects, such as *D. melanogaster* and *Anopheles. gambiae*, and functional studies have revealed a reduction in the responses to odorant stimulation by arrestin mutants (20, 21). However, the underlying mechanisms remain cloudy. Herein, we found that BmOR3 directly recruited arrestin *via* the intracellular N terminus, which mediated BmOR3 internalization. Arrestin senses and interacts with the negatively charged residues in the C tails of GPCRs by different phosphate-binding pockets (37, 38). Accordingly, we showed that the potential phosphorylation sites within the N terminus of BmOR3 are important for both arrestin recruitment and receptor internalization. Notably, the transmembrane cores of GPCRs participate in arrestin activation (53, 54). Whether a similar mechanism exists in insect PRs requires further investigation. Previous studies have revealed that the temporal dynamics of insect ORs differ for odor stimuli of different quality and quantity, and this temporal coding contributes to odor discrimination (9). Therefore, arrestin-mediated OR internalization has added a new layer to the complexity of the spatiotemporal coding of odors and pheromones by insect OR systems. Future strategies targeting arrestin might be helpful in controlling olfactory-based behaviors in insects.

In addition to its roles in the regulation of BmOR3 internalization, we have shown that arrestin contributes to BmOR3–BmOrco complex formation. This is essential for the ionotropic functions of the BmOR3–BmOrco complex because the downstream calcium response was specifically mediated by extracellular Ca^2+^ influx. Whether the structural and functional integrity of BmOR3–BmOrco is regulated by arrestins through downstream effector-induced modulation (*e.g.*, kinase-mediated phosphorylation) or by direct physical scaffolding remains unknown. Our current results suggested arrestin mediates activation of kinases including ERK, SRC, and AKT, which might be functionally involved in the regulation of BmOR3–BmOrco activity. In addition, our recent studies have demonstrated the formation of GPCR–ion channel complexes, such as AT1R–TRPC3 and ADGRG2–CFTR, which are scaffolded by β-arrestin-1 (36, 40). Therefore, it is also possible that arrestin directly bridges PRs to Orco to initiate acute cation influx. Nevertheless, the precise underlying mechanism still needs further investigation. Finally, it is worth noting that the results in the present study were obtained from the heterologous system by overexpressing insect PR and Orco in mammalian cells, which may not reflect the native signaling properties *in vivo*. Therefore, the mechanism underlying insect PR activation proposed in this study is expected to be further verified in insects.

In conclusion, we found that the agonist-stimulated active conformational changes of BmOR3 were transmitted to the coreceptor BmOrco through key interactions between residues at S7 from both receptors, which might be a common mechanism underlying the interactions between insect PR and Orco. We provided direct evidence that BmOR3 could couple to both G protein and arrestin. Whereas the Gi pathway is dispensable for complex formation between BmOR3 and BmOrco and the ionotropic functions of BmOR3, arrestin plays critical roles in these two physiological processes.

## Experimental procedures

### Materials

The GloSensor cAMP assay reagent (E1290), Rluc substrate Coelenterazine h (S2011), and Nluc substrate furimazine (N1661) were purchased from Promega. TC-FlAsH II In-Cell Tetracysteine Tag Detection Kit (T34561) was purchased from Thermo Scientific. Bombykal was synthesized in our own lab. All the other reagents or chemicals were purchased from Sigma-Aldrich unless otherwise specified.

### Constructs

BmOR3 and BmOrco coding sequences were subcloned in pcDNA3.1 expression vector, respectively. BmOR3–β2AR and BmOrco–β2AR chimeras were constructed by PCR-directed homologous DNA recombination. The other mutants of BmOR3 or BmOrco used in the present study were generated using QuikChange Mutagenesis Kit (StrataGene). CalfluxVTN was synthesized and cloned into pcDNA3.1 vector according to previous report (23). ERK, SRC, AKT, and JNK3 BRET sensors were constructed by fusing the YFP coding sequence in frame at the N terminus of respective kinases. All the constructs and mutations were verified by DNA sequencing. The primers used in our study are shown in Table S1.

### Cell culture and transfection

The HEK293 cells were obtained from the Cell Resource Center of Shanghai Institute for Biological Sciences (Chinese Academy of Sciences). The cells were cultured in Dulbecco's modified Eagle's medium (DMEM) supplemented with 10% fetal bovine serum, 100 IU/ml penicillin, and 100 μg/ml streptomycin at 37 °C in a humidified atmosphere containing 5% CO_2_. Cells used in the present study were transfected using Lipofectamine 2000 unless otherwise specified.

### Cell-surface ELISA

HEK293 cells were transfected with C-terminal Flag-tagged BmOR3 or BmOrco or their mutants in 24-well plates. After incubation at 37 °C for 48 h, the cells were fixed with 4% (w/v) formaldehyde for 5 min followed by incubation in the blocking solution (5% bovine serum albumin in Dulbecco's phosphate buffered saline) for 1 h at room temperature (RT). The cells were incubated overnight with an anti-Flag primary antibody (Sigma-Aldrich, Cat# F1804, 1:1000) at 4 °C followed by incubation with a horseradish peroxidase–conjugated secondary anti-rabbit antibody (Thermo Fisher, Cat# A-21235, 1:5000) for 1 h at RT. After washing with Dulbecco's phosphate buffered saline, the tetramethyl benzidine solution was added and the color reaction was stopped by adding an equal volume of 0.25 M HCl solution. The optical density of each well was measured at 450 nm using the TECAN luminescence counter (Infinite M200 Pro NanoQuant). The optical density was plotted against the transfecting amounts of respective plasmids to determine the relative expression levels of each receptor or mutants.

### CalfluxVTN Ca^2+^ assay

HEK293 cells were cotransfected with plasmids encoding WT BmOR3 and BmOrco or their mutants, or AT1R together with CalfluxVTN probe. 24 h after transfection, cells were redistributed into 96-well flat-bottomed microplates. After another 24 h, cells were incubated with conventional buffer with Ca^2+^ (135 mM NaCl, 5 mM KCl, 1 mM CaCl_2_, 5 mM D-glucose, 10 mM Hepes; pH = 7.4) or with Ca^2+^-free buffer (135 mM NaCl, 5 mM KCl, 1 mM EGTA, 5 mM D-glucose, 10 mM Hepes; pH = 7.4), and stimulated with different concentrations of Bombykal (or AngII). The BRET ratio between Venus and Nluc was measured before (baseline) and after the addition of the Nluc substrate furimazine (5 μM) using a Mithras LB 940 multimode reader (Berthold Technologies). The BRET signal was calculated as ratio of emission of Venus (530 nm) to Nluc (460 nm). The baseline value was subtracted from the bombykal-stimulated BRET signal to obtain the ΔBRET value.

### BmOR3–BmOrco BRET assay

HEK293 cells were cotransfected with the N-terminal Rluc-tagged WT BmOR3 together with N-terminal YFP-fused BmOrco plasmids or their mutants. Twenty-four hours after transfection, cells were distributed into a 96-well microplate. After another 24 h, the cells were stimulated with different concentrations of bombykal for 5 min. BRET between RLuc and YFP was measured using a Mithras LB 940 multimode reader after the addition of the RLuc substrate coelenterazine h (5 μM). The BRET signal was calculated as the ratio of emission of YFP (530 nm) to RLuc (485 nm). For the saturation BRET between Rluc-BmOrco and YFP-BmOR3, HEK293 cells were transfected with a fixed amount of Rluc-BmOrco and increasing amounts of YFP-BmOR3. The BRET signal between RLuc and YFP was directly measured after the addition of the substrate coelenterazine h (5 μM).

### FlAsH-BRET assay

BmOR3-FlAsH constructs were generated by fusing a Rluc moiety to the N terminus of BoOR3 and inserting a TC-tag (CCPGCC) into a specific position at different loops of BmOR3, including T116 (ICL1), N266 (ICL2), E380 (ICL3), V381 (ICL3), H406 (loop7a-7b), and L410 (loop7a-7b). BmOrco-FlAsH constructs were generated by fusing an Rluc moiety to the N terminus of BmOrco and inserting a TC-tag (CCPGCC) into the specific position at different loops of BmOrco, including S112 (ICL1), A274 (ICL2), S414 (ICL3), C415 (ICL3), M439 (7a-7b loop), and A444 (7a-7b loop). HEK293 cells were transfected with BmOR3-FlAsH probes or WT BmOR3 and BmOrco-FlAsH probes. 48 h after transfection, the cells were labeled with 2.5 μM FlAsH-ETD2 solution from a TC-FlAsH II In-Cell Tetracysteine Tag Detection Kit (Thermo Scientific) according to the manufacturer's instructions. The cells were stimulated with bombykal, and the BRET signal between Rluc and FlAsH acceptor was measured after the addition of RLuc substrate coelenterazine h (5 μM) using a Mithras LB 940 microplate reader (Berthold Technologies). The BRET signal was determined by calculating the ratio of the FlAsH emission (530 nm) over the RLuc emission (485 nm). The change in the BRET signal due to bombykal stimulation was reported as ΔBRET.

### GloSensor cAMP assay

The GloSensor cAMP assay was performed as previously described (40, 55, 56). Briefly, HEK293 cells were transfected with the BmOR3 plasmid or empty vector pcDNA3.1 together with the GloSensor plasmid. 24 h after transfection, cells were distributed into 96-well microplates at a density of 50,000 cells/well. After another 24 h, the cells were incubated with DMEM containing 2% (v/v) GloSensor cAMP reagent for 2 h at 37 °C. Cells were then treated with varying concentrations of bombykal followed by the stimulation of 1 μM forskolin. The luminescence intensity was measured using an EnVision multi-label microplate detector (PerkinElmer).

### G protein recruitment

Gi1-Rluc, Gi2-Rluc, and Gi3-Rluc plasmids were constructed according to previous report (30). HEK293 cells were transiently cotransfected with N-terminal YFP-tagged BmOR3 together with Gi-Rluc plasmids. Twenty-four hours after transfection, cells were distributed into a 96-well microplate. After another 24 h, the cells were stimulated with an increasing amount of bombykal for 2 min. The light emission of Rluc (485 nm) and YFP (530 nm) was measured after the addition of the Rluc substrate coelenterazine h (5 μM) using a Mithras LB 940 multimode reader (Berthold Technologies). The BRET signal was calculated as the ratio of emission of YFP to Rluc. The net BERT value was calculated by subtracting the BRET signal obtained in the cells transfected with Rluc-Gi plasmids only.

### G protein dissociation assay

The Gi, Gq, Go, Gs, and Gz dissociation BRET probes were from the TRUPATH kit, which was a gift from Bryan Roth (Addgene kit #1000000163 (31)). HEK293 cells were cotransfected with BmOR3 along with specific G protein BRET probes. After 24 h, the cells were reseeded in 96-well microplates and incubated for another 24 h. The cells were washed twice with Hank's balanced salt solution and stimulated with bombykal at different concentrations for 2 min. The light emission of Rluc8 (400 nm) and GFP10 (510 nm) was measured after the addition of the substrate coelenterazine 400a (5 μM) using a Mithras LB 940 multimode reader (Berthold Technologies). The BRET signal was calculated as the ratio of light emission at 510/400 nm.

### β-Arrestin recruitment

HEK293 cells were transiently cotransfected with the N-terminal YFP-tagged BmOR3 together with N-terminal Rluc-fused β-arrestin-1, β-arrestin-2, or BmArr plasmids. Twenty-four hours after transfection, cells were distributed into a 96-well microplate and incubated for additional 24 h. The cells were stimulated with varying concentrations of bombykal for 10 min, and the BRET signal between YFP (530 nm) and Rluc (485 nm) was measured after the addition of the Rluc substrate coelenterazine h (5 μM) using a Mithras LB 940 multimode reader (Berthold Technologies). The BRET signal was calculated as the ratio of emission of YFP to Rluc.

### Arrestin-kinase BRET assay

HEK293 cells were transiently cotransfected with the WT BmOR3, BmArr-Rluc, and YFP-tagged ERK, SRC, AKT, or JNK3, or with the WT AT1R, β-arr2-Rluc, and YFP-tagged ERK, SRC, AKT, or JNK3. Twenty-four hours after transfection, cells were distributed into a 96-well microplate. After another 24 h, the cells were stimulated with 1 μM bombykal (for BmOR3-transfected cells) or 1 μM AngII for 10 min (for AT1R-transfected cells). BRET between RLuc and YFP was measured using a Mithras LB 940 multimode reader after the addition of the RLuc substrate coelenterazine h (5 μM). The BRET signal was calculated as the ratio of emission of YFP (530 nm) to RLuc (485 nm).

### Internalization assay

BRET-based internalization assay was performed as previously described (57). Briefly, HEK293 cells treated with Ctrl or β-arrestin-1/2 siRNA, or pretransfected with BmArr, were transiently transfected with N-terminal Rluc-fused BmOR3 and plasma membrane marker Lyn-YFP. Twenty-four hours after transfection, the cells were detached and distributed into a 96-well microplate. After another 24-h incubation at 37 °C, the cells were stimulated with vehicle or 1 μM bombykal at 37 °C for 20 min. Rluc substrate coelenterazine h was added at a final concentration of 5 μM before light emissions of YFP and Rluc were recorded using a Mithras LB 940 microplate reader (Berthold Technologies). The BRET signal was calculated as the ratio of emission of YFP to Rluc.

### Western blotting

HEK293 cells transfected with BmOR3 in the absence or presence of βarrestin1/2 siRNA were starved for 12 h at 37 °C before stimulated with 1 μM bombykal for 15 min. The cells were lysed in ice-cold lysis buffer (50 mM Tris-HCl, pH 7.4, 150 mM NaCl, 1 mM NaF, 1% Triton, 2 mM EDTA, 1% NP-40, 1% protease and phosphatase inhibitor cocktail), and the supernatant was collected after centrifugation. Proteins were separated by PAGE and transferred to PVDF membrane. After blocking in PBS containing 5% bovine serum albumin and 0.1% Tween-20, the membrane was incubated with the primary antibody at 4 °C overnight, followed by incubation with HRP conjugate–secondary antibody at RT for an hour. The signals were detected with the ECL system (Thermo Fisher Scientific). Primary antibodies were as follows: anti-ERK (Santa Cruz, Cat. No. sc-154); anti-pERK (Proteintech, Cat. No. 15361-1-AP); anti-SRC (Cell Signaling Technology, Cat. No. 2109S); anti-pSRC(Y416) (Cell Signaling Technology, Cat. No. 2101S); anti-beta actin (ORIGENE, Cat. No. TA811000); anti-βarrestin1(Proteintech, Cat. No. 15361-1-AP); anti-βarrestin2 (Proteintech, Cat. No. 10171-1-AP).

### Statistical analysis

All data in this study are presented as the mean ± SEM from at least three independent experiments. Statistical comparisons were performed using Student's *t* test or one-way ANOVA with GraphPad Prism 8.0 software. Significant differences were accepted when *p* values were below 0.05.

## Data availability

All remaining data are contained within the article.

## Supporting information

This article contains supporting information.

## Conflict of interest

The authors declare that they have no conflicts of interest with the contents of this article.
