# Supplementary material for: An ionic lock and a hydrophobic zipper mediate the coupling between an insect pheromone receptor BmOR3 and downstream effectors
Source: J Biol Chem. 2021 Sep 2;297(4):101160. doi: 10.1016/j.jbc.2021.101160 (PMC8477192; doi:10.1016/j.jbc.2021.101160)
Supplement: Figures S1–S4 and Table S1 [file mmc1.docx]

**Supplementary Materials for**

**An ionic lock and a hydrophobic zipper mediate the coupling between an insect pheromone receptor BmOR3 and downstream effectors**

Jing-Yu Lin^#1^, Zhao Yang^#2^, Chan Yang^#3^, Ji-Xiang Du^#2^, Fan Yang^2^, Jie Cheng^1^, Wei Pan^1^, Shi-Jie Zhang^1^, Xu Yan^2^, Jia Wang^2^, Jin Wang^4^, Lu Tie^5^, Xiao Yu*^1^, Xin Chen*^6^ and Jin-Peng Sun*^2,3^

^1^Key Laboratory Experimental Teratology of the Ministry of Education and Department of Physiology, School of Basic Medical Sciences, Shandong University, 250012 Jinan, China

^2^Key Laboratory Experimental Teratology of the Ministry of Education and Department of Biochemistry and Molecular Biology, School of Basic Medical Sciences, Shandong University, 250012 Jinan, China

^3^Department of Physiology and Pathophysiology, School of Basic Medical Sciences, Peking University, Key Laboratory of Molecular Cardiovascular Science, Ministry of Education, 100191 Beijing, China

^4^Department of Pharmacology, School of Basic Medical Sciences, Shandong University, 250012 Jinan, China

^5^Department of Pharmacology, School of Basic Medical Sciences, Peking University, 100191 Beijing, China

^6^Department of Medicinal Chemistry, School of Pharmaceutical Engineering and Life Science, Changzhou University, 213164 Changzhou, Jiangsu, China.

# These authors contributed equally to this work.

* Corresponding authors: Jin-Peng Sun (sunjinpeng@sdu.edu.cn.)

Xin Chen ([xinchen@cczu.edu.cn](file:///C:\Users\yangchan\Desktop\北医2019\盛超龚正文章\CTR-初次投稿-07-07\Documents\WeChat%20Files\wxid_fa3m0593tflp22\FileStorage\File\2019-07\xinchen@cczu.edu.cn))

Xiao Yu ([yuxiao@sdu.edu.cn](mailto:yuxiao@sdu.edu.cn))


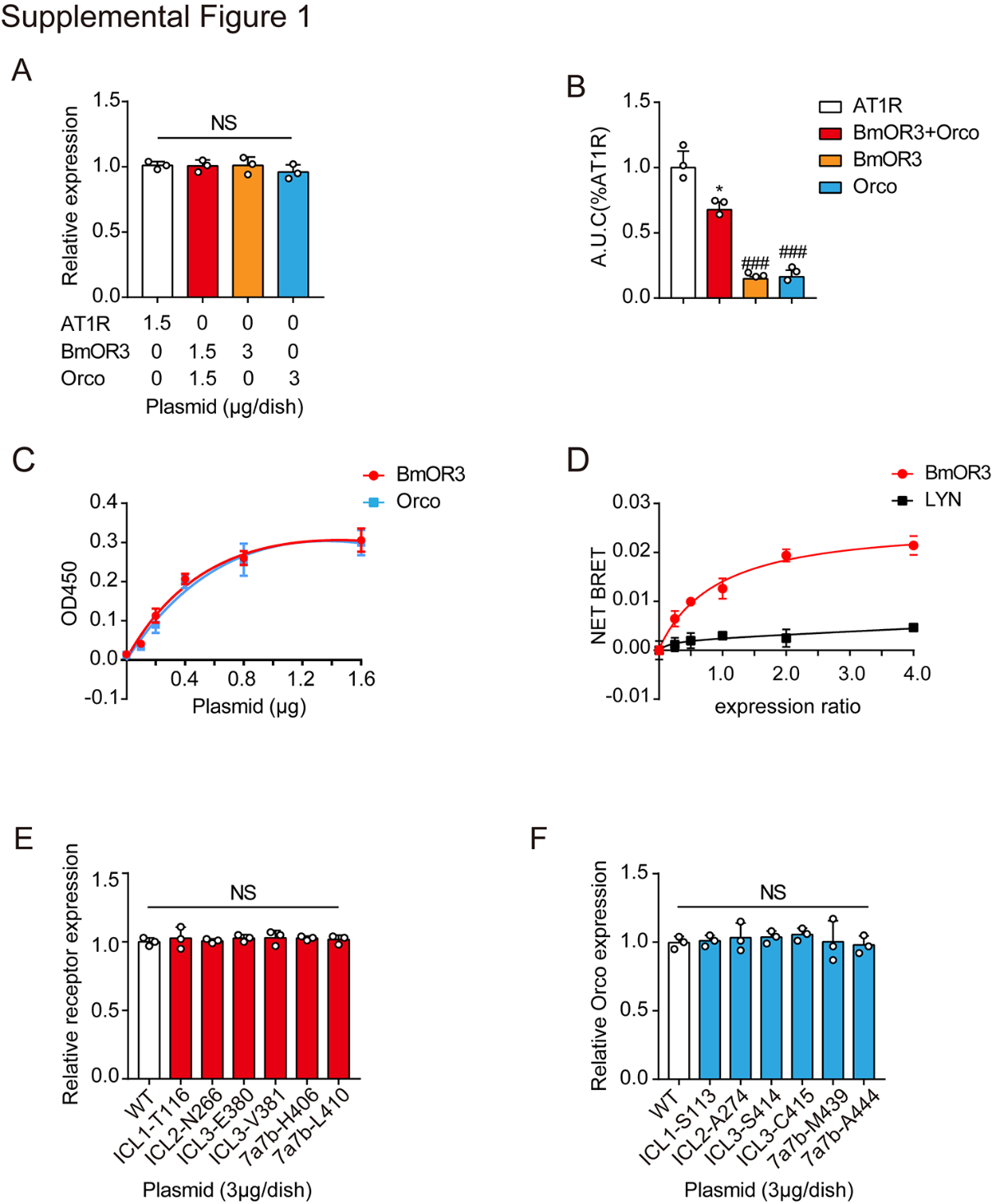


**Supplemental Figure 1 Constitutive and bombykal-stimulated functional coupling between BmOR3 and BmOrco.**

(A) Cell surface ELISA assay showing similar expression levels of AT1R, BmOR3, BmOrco, or BmOR3-BmOrco combination in HEK293 cells.(B) Bombykal- or AngII-induced calcium influx in CalfuluxVTN-expressing HEK293 cells transfected with AT1R, BmOR3, BmOrco or both BmOR3 and BmOrco. Data are correlated to Fig. 1B.(C) Cell surface ELISA assay showing similar expression patterns of BmOR3 and BmOrco in HEK293 cells.(D) Saturation BRET signal between BmOrco-Rluc and BmOR3 in HEK293 cells. The lyn-YFP was used as the negative control.(E-F) Cell surface ELISA assay showing similar expression levels of WT BmOR3 (E) or BmOrco (F) and their mutants. (A-B) ns, no significant difference; *p < 0.05. HEK293 cells transfected with BmOR3 or BmOrco were compared with those transfected with AT1R. ^###^p<0.001. Cells transfected with BmOR3 or BmOrco alone were compared with cells transfected with both BmOR3 and BmOrco. (E-F) ns, no significant difference. HEK293 cells transfected with BmOR3 or BmOrco mutants were compared with those transfected with wide-type receptor. Data are shown as the mean ± SD of at least three independent experiments. Data statistics were analyzed using one-way ANOVA with Dunnett’s post hoc test.


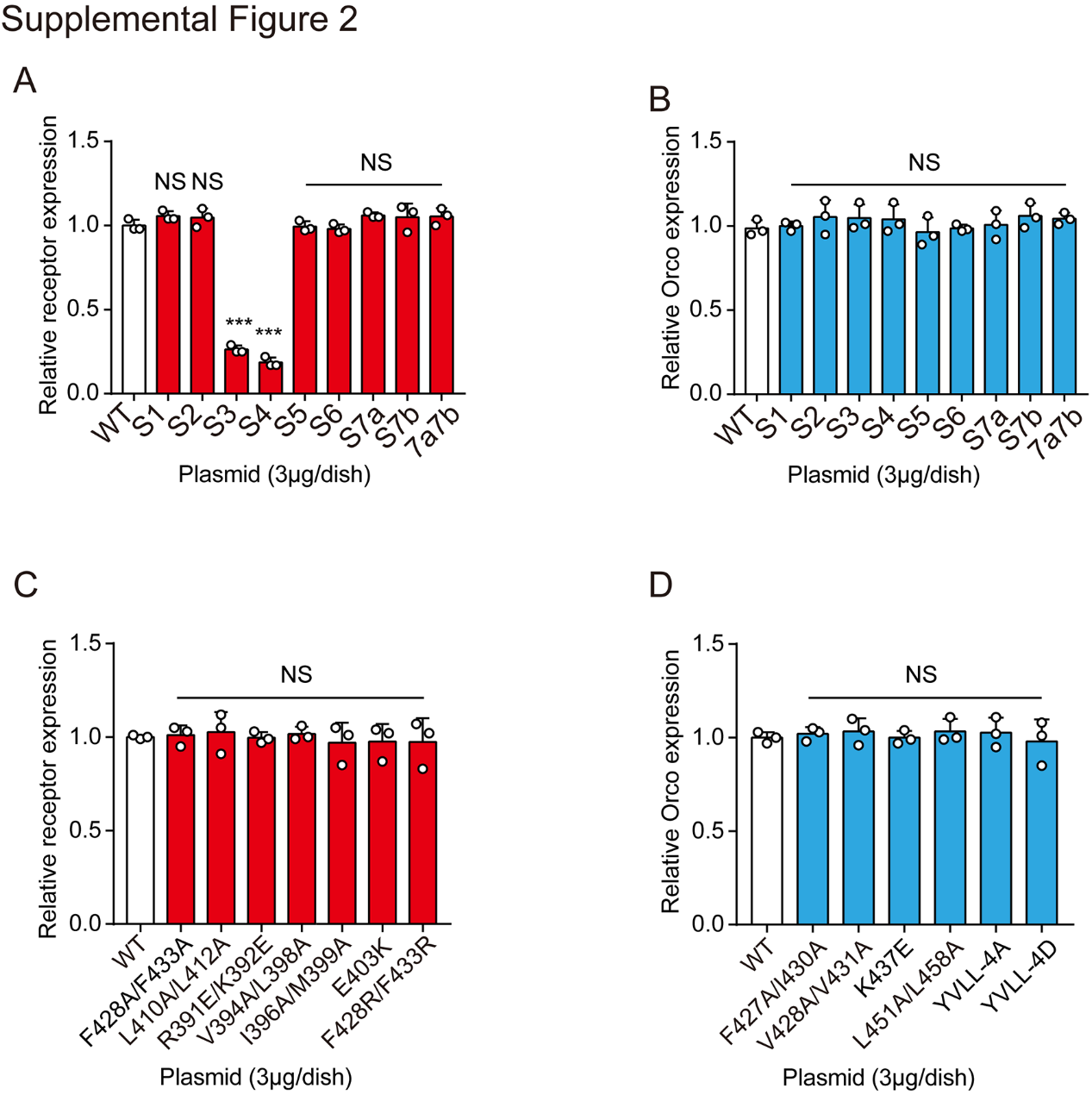


**Supplemental Figure 2 Expression levels of BmOR3 and BmOrco mutants in HEK293 cells.**

(A-B) Cell surface ELISA assay showing similar expression levels of WT BmOR3 and chemiras (A) or WT BmOrco and chimeras (B) in HEK293 cells. Notably, the substitution of S3 or S4 in BmOR3 caused the deficiency of receptor expression. (C-D) Cell surface ELISA assay showing similar expression levels of WT BmOR3 and mutants (A) or WT BmOrco and mutants (B) in HEK293 cells. (A-D) ns, no significant difference; ***p < 0.001. HEK293 cells transfected with BmOR3 or BmOrco chimeras or mutants were compared with those transfected with wide-type receptor. Data are shown as the mean ± SD of at least three independent experiments. Data statistics were analyzed using one-way ANOVA with Dunnett’s post hoc test.


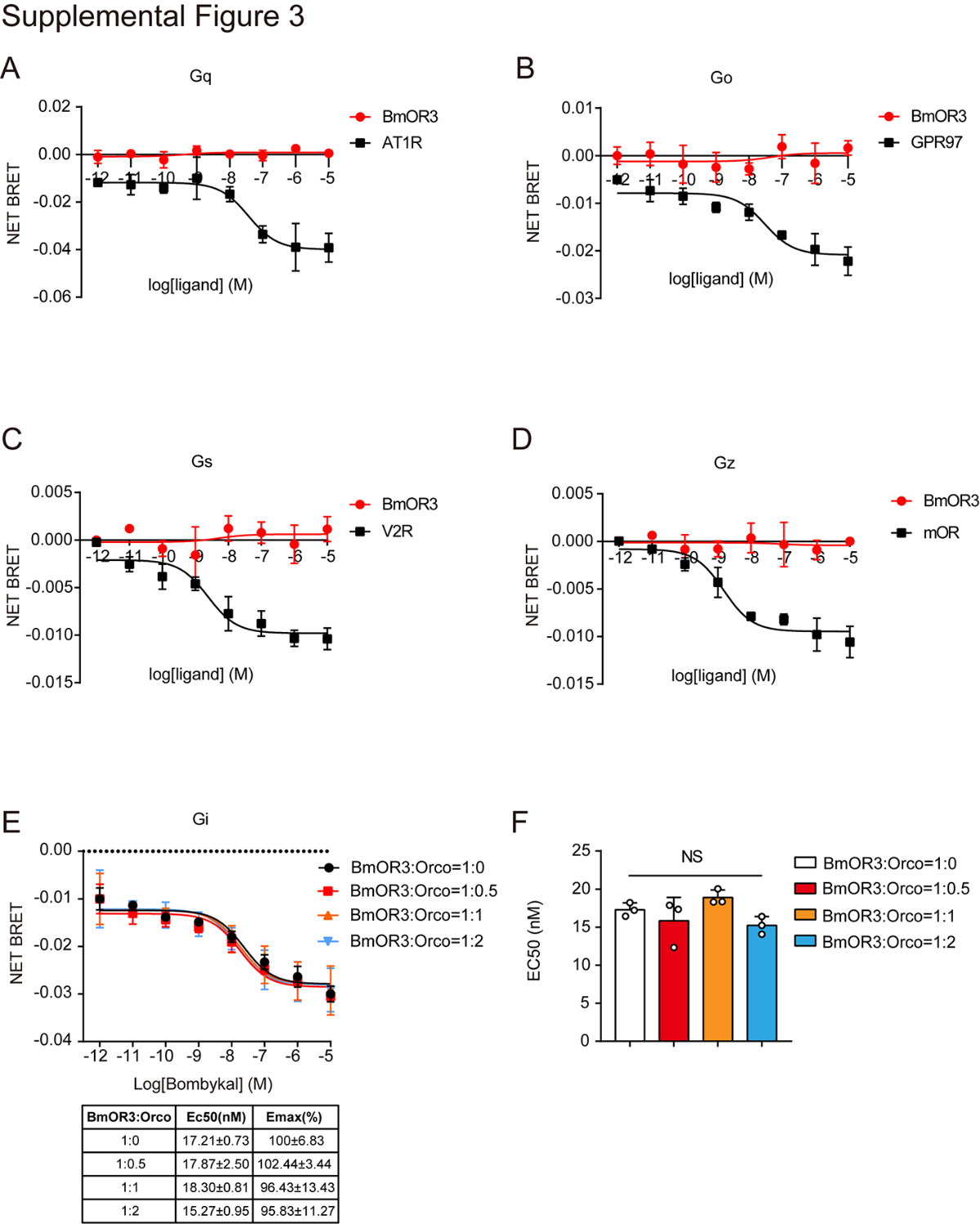


**Supplemental Figure 3 G protein signaling properties of BmOR3.**

(A) Bombykal or AngII-induced dose-dependent Gq activation in HEK293 cells transfected with BmOR3 or AT1R measured by G protein dissociation BRET assay. (B) Bombykal or cortisol-induced dose-dependent Go activation in HEK293 cells transfected with BmOR3 or GPR97 measured by G protein dissociation BRET assay. (C) Bombykal or AVP-induced dose-dependent Gs activation in HEK293 cells transfected with BmOR3 or V2R measured by G protein dissociation BRET assay. (D) Bombykal or DAMGO-induced dose-dependent Gz activation in HEK293 cells transfected with BmOR3 or MOR measured by G protein dissociation BRET assay. (E) Bombykal-induced dose-dependent Gi activation in HEK293 cells transfected with a fixed amount of BmOR3 and increasing amounts of BmOrco. (F) The potency of bombykal-induced Gi activation in HEK293 cells transfected with a fixed amount of BmOR3 and increasing amounts of BmOrco. Data are correlated to Fig. S3E.(F) ns, no significant difference; HEK293 cells transfected with BmOR3 and BmOrco were compared with those transfected only with BmOR3. Data are shown as the mean ± SD of at least three independent experiments. Representative curves from three independent experiments were shown. Data statistics were analyzed using one-way ANOVA with Dunnett’s post hoc test.


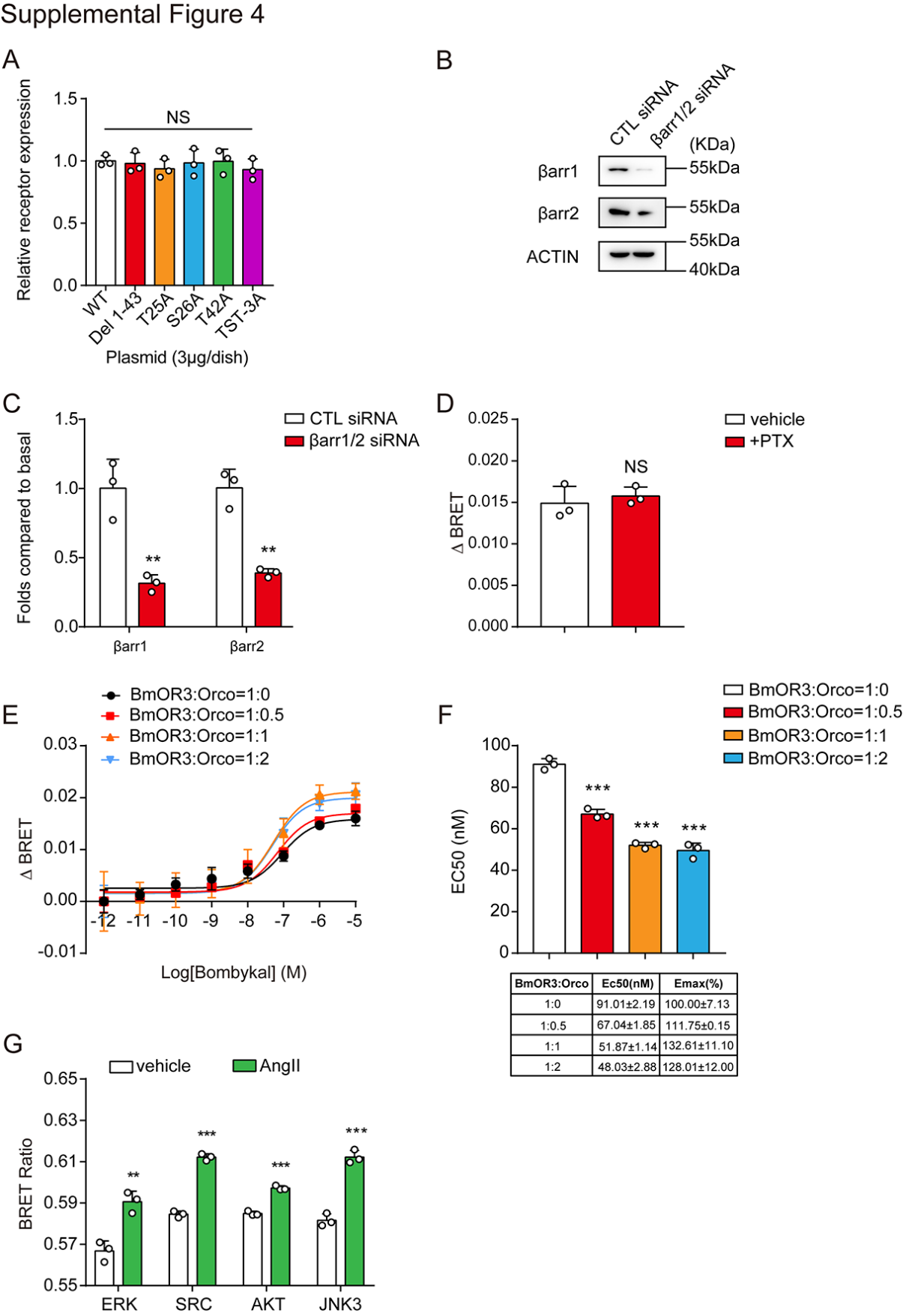


**Supplemental Figure 4 BmOR3 interacts with arrestins in Gi-independent but BmOrco-dependent manner**

(A) Cell surface ELISA assay showing similar expression levels of WT BmOR3 and mutants in HEK293 cells. (B-C) Representative blots (B) and quantification (C) of the βarr1 and βarr1 protein levels in HEK293 cells transfected with control siRNA or βarr1/2 siRNA (D) Bombykal-induced BmArr recruitment to BmOR3 in HEK293 cells pretreated with control vehicle or PTX measured by BRET assay. (E) Bombykal-induced dose-dependent BmArr recruitment to BmOR3 in HEK293 cells transfected with a fixed amount of BmOR3 and increasing amounts of BmOrco. (F) The potency of bombykal-induced BmArr recruitment to BmOR3 in HEK293 cells transfected with a fixed amount of BmOR3 and increasing amounts of BmOrco. Data are correlated to Fig. S4E. (G) AngII-induced interaction of Rluc-β-arr2 with YFP-tagged ERK, SRC, AKT or JNK3 in AT1R-expressing HEK293 cells. (A) ns, no significant difference. HEK293 cells transfected with BmOR3 mutants were compared with those transfected with WT BmOR3. (C) **p < 0.01. HEK293 cells transfected with βarr1/2 siRNA were compared with those transfected with control siRNA. (D) ns, no significant difference. HEK293 cells treated with PTX were compared with those treated with vehicle. (F) ***p < 0.001. HEK293 cells transfected with BmOR3 and BmOrco were compared with those transfected only with BmOR3. (G) **p < 0.01; ***p < 0.001. HEK293 cells stimulated with AngII were compared with those treated with vehicle. Data are shown as the mean ± SD of at least three independent experiments. Data statistics were analyzed using one-way ANOVA with Dunnett’s post hoc test.

**Supplementary Table 1. List of primers used in the present study.**

Primer with suffix “-F” means forward primer, and primers with suffix “-R” means reverse primers.

| **Primers** | **Sequence** |
| --- | --- |
| AR-S2-F | 5’-ACCAACTACTTCATCACTTCACTGG-3’ |
| AR-S2-R | 5’-TTTCATAAGAATATGGGCGGCCCCAA-3’ |
| AR-S3-F | 5’-AACTTCTGGTGCGAGTTTTGGACTTCC-3’ |
| AR-S3-R | 5’-TGAAGTAATGGCAAAGTAGCGATCCAC-3’ |
| AR-S4-F | 5’-ACCAAGAATAAGGCCCGGGTG-3’ |
| AR-S4-R | 5’-CATCTGAATGGGCAAGAAGGAGGTA-3’ |
| AR-S5-F | 5’-AACCAAGCCTATGCCATTGCCTCTT-3’ |
| AR-S5-R | 5’-CTCAGATTTGTCAATCTTCTGGAGC-3’ |
| AR-s6-F | 5’-TCCAAGTTCTGCTTGAAGGAGCACAAAGC-3’ |
| AR-S6-R | 5’-CTGGATCACATGCACAATGTTAACGATG-3’ |
| AR-S7-F | 5’-CGTAAGGAAGTTTACATCCTCCTAAATTGG-3’ |
| AR-S7-R | 5’-GCAGTAGATAAGGGGATTGAAACCAGAATTG-3’ |
| B3TH-S1-F | 5’-CCATATTCTTATGAAAGACGTGCTGGGCTTCTTCGAGCTGGGC-3’ |
| B3TH-S1-R | 5’-TGATGAAGTAGTTGGTGGGGGCGCACTCGTACTTGCTGCCGGT-3’ |
| B3TH-S2-F | 5’-CTTTGCCATTACTTCATGGAACCCCAACTACCACAAGGTGGTG-3’ |
| B3TH-S2-R | 5’-CTCGCACCAGAAGTTGCCCAGCTCGAAGAAGCCCAGCACGTC-3’ |
| B3TH-S3-F | 5’-CTTGCCCATTCAGATGCCCATGTACAACAACTACCGCCAGGGC-3’ |
| B3TH-S3-R | 5’-GGGCCTTATTCTTGGTGTGCACGCGGCGGTGGGTCTTCATGGC-3’ |
| B3TH-S4-F | 5’-GATTGACAAATCTGAGATCCTGATCCACAACCTGCTGACCTTC-3’ |
| B3TH-S4-R | 5’-TGGCATAGGCTTGGTTGATGTAGCTGGCGAACCAGTTGAACAC-3’ |
| B3TH-S5-F | 5’-TGTGCATGTGATCCAGCACCAGGTGAGCGGCTGCCTGCTGCTG-3’ |
| B3TH-S5-R | 5’-TTTGTGCTCCTTCAAGCAGTAGTCGATGCACTGGCGCAGGCGGTC-3’ |
| B3TH-S6-F | 5’-TCCCCTTATCTACTGCGTGGGCACCGTGACCGGCCGCCTGAAG-3’ |
| B3TH-S6-R | 5’-GTAAACTTCCTTACGGCGCACCAGGGCGGCGGTGTTCAGCTGG-3’ |
| B-7a-F | 5’-TTCAGGATTGCCTTCCAGGAGCAGGAGCCCCTGCACGTGAACGCCCTGG-3’ |
| B-7a-R | 5’-GGCAATCCTGAAATCTGGGCTCACCTCGTACACGGCGTCCTTCAGGCGG-3’ |
| B-7b-F | 5’-GTGCATGTGATCCAGACCGTGAGCGAGTAAGAATTCTGCAG-3’ |
| B-7b-R | 5’-CAAGCAGAACTTGGACACCTTGGCCAGGCCCAGGGCGTTCAC-3’ |
| B3-67a-JD-F | 5’-GGTGGAGGAGGTTCAGAGTACATGGACACCAGCAACCGCAAG-3’ |
| B3-67a-JD-R | 5’-TGAACCTCCTCCACCGGCGTCCTTCAGGCGGCCGGTCACGGT-3’ |
| B3-7a7b-JD-F | 5’-GGTGGAGGAGGTTCAGGCGTGCAGAGCATGGCCGCCATCCTGA-3’ |
| B3-7a7b-JD-R | 5’-TGAACCTCCTCCACCCACGTTCATCAGGAAGATGGCCACGGTC-3’ |
| BoTH-S1-F | 5’-CCATATTCTTATGAAAAAGGACGAAGTCAACGAGCTCACGGCA-3’ |
| BoTH-S1-R | 5’-TGATGAAGTAGTTGGTCTGCGTCTTGACCTTGGTCATCATTTG-3’ |
| BoTH-S2-F | 5’-CTTTGCCATTACTTCATGGAATCAGTCCAACAGCCATCCGTTG-3’ |
| BoTH-S2-R | 5’-CTCGCACCAGAAGTTGTCCTTGTATTGTGCCATGTTGATGCC-3’ |
| BoTH-S3-F | 5’-CTTGCCCATTCAGATGTTCGGTGAATCTGTCCGTATGATAGCC-3’ |
| BoTH-S3-R | 5’-GGGCCTTATTCTTGGTCAACGGATGGCTGTTGGACTGATTCCA-3’ |
| BoTH-S4-F | 5’-GATTGACAAATCTGAGCTAAGCGCTGCTTTGGACACCTACCGT-3’ |
| BoTH-S4-R | 5’-TGGCATAGGCTTGGTTACTCATTGTCTTAAATGGATACCACGC-3’ |
| BoTH-S5-F | 5’-TGTGCATGTGATCCAGATAAACGGTATAAACGTGTATGCATTC-3’ |
| BoTH-S5-R | 5’-TTTGTGCTCCTTCAAGCACAAACCGTTAGGATTGTTCTCCGCATT-3’ |
| BoTH-S6-F | 5’-CCCCTTATCTACTGCTGTCAGTGGTACGACGGTTCCGAGGAGGC-3’ |
| BoTH-S6-R | 5’-GTAAACTTCCTTACGCACGTTTATACCGTTTATCTTAGTCGCCTGG-3’ |
| Bo-7a-F | 5’-TTCAGGATTGCCTTCCAGGAGCAGAAGGCGATGACGATCTCTGGAGC-3’ |
| Bo-7a-R | 5’-GGCAATCCTGAAATCTGGGCTACACGAGTAGGCGGCCTCCATAACCG-3’ |
| Bo-7b-F | 5’-GTGCATGTGATCCAGCAACTGAAGTAGCTCGAGTCTAGAGGG-3’ |
| Bo-7b-R | 5’-CAAGCAGAACTTGGAAACATTGAAGAATTTCGCTCCAGAGATC-3’ |
| Bo-67a-JD-F | 5’-GGTGGAGGAGGTTCATACGACGGTTCCGAGGAGGCGAAGAC-3’ |
| Bo-67a-JD-R | 5’-TGAACCTCCTCCACCGGCGGCCTCCATAACCGATGAGCTCTCC-3’ |
| Bo-7a7b-JD-F | 5’-GGTGGAGGAGGTTCATCACTGGATTTGTTCGCTTCGGTGCTG-3’ |
| Bo-7a7b-JD-R | 5’-TGAACCTCCTCCACCGCATTGTTGGCAGACGATCTGCACGAAC-3’ |
| BmOR3-F428A/F433A-F | 5’-AGCGCCAGCTACTTCACCGCCCTGCGCACCGTGAGCGAG-3’ |
| BmOR3-F428A/F433A-R | 5’-GGTGAAGTAGCTGGCGCTGGTCTTCAGGATGGCGGCCATGCTC-3’ |
| BmOR3-L410A/L412A-F | 5’-CCGCGGGCGCGGCCAAGGTGGGCGTGCAGAGCATGGCCGCCATC-3’ |
| BmOR3-L410A/L412A-R | 5’-CTTGGCCGCGCCCGCGGCGTTCACGTGCAGGGGCTCCTGCAC-3’ |
| BmOR3-R391A/K392E-F | 5’-CACCAGCAACCGCAAGACCGTGGCCATCTTCCTGATGAACGTG-3’ |
| BmOR3-R391A/K392E-R | 5’-TGGCCACGGTCTCCTCGTTGCTGGTGTCCATGTACTCCCAG-3’ |
| BmOR3-V394A/L398A-F | 5’-ACCGCGGCCATCTTCGCGATGAACGTGCAGGAGCCCCTGCAC-3’ |
| BmOR3-V394A/L398A-R | 5’-CATCGCGAAGATGGCCGCGGTCTTGCGGTTGCTGGTGTCCATG-3’ |
| BmOR3-I396A/M399A-F | 5’-TGGCCGCCTTCCTGGCGAACGTGCAGGAGCCCCTGCAC-3’ |
| BmOR3-I396A/M399A-R | 5’-CGTTCGCCAGGAAGGCGGCCACGGTCTTGCGGTTGCTG-3’ |
| BmOR3-E403K-F | 5’-CTGATGAACGTGCAGAAGCCCCTGCACGTGAACGCCCTG-3’ |
| BmOR3-E403K-R | 5’-TCACGTGCAGGGGCTTCTGCACGTTCATCAGGAAGATGGCCAC-3’ |
| BmOR3-F428A/F433A-F | 5’-AGCGCCAGCTACTTCACCGCCCTGCGCACCGTGAGCGAG-3’ |
| BmOR3-F428A/F433A-R | 5’-GGTGAAGTAGCTGGCGCTGGTCTTCAGGATGGCGGCCATGCTC-3’ |
| BmOR3-L410A/L412A-F | 5’-CCGCGGGCGCGGCCAAGGTGGGCGTGCAGAGCATGGCCGCCATC-3’ |
| BmOR3-L410A/L412A-R | 5’-CTTGGCCGCGCCCGCGGCGTTCACGTGCAGGGGCTCCTGCAC-3’ |
| BmOR3-R391A/K392E-F | 5’-CACCAGCAACCGCAAGACCGTGGCCATCTTCCTGATGAACGTG-3’ |
| BmOR3-R391A/K392E-R | 5’-TGGCCACGGTCTCCTCGTTGCTGGTGTCCATGTACTCCCAG-3’ |
| BmOR3-V394A/L398A-F | 5’-ACCGCGGCCATCTTCGCGATGAACGTGCAGGAGCCCCTGCAC-3’ |
| Orco-F427A/I430A-F | 5’-AGACGGCCGTGCAGGCCGTCTGCCAACAATGCCAGAAGGCGATG-3’ |
| Orco-F427A/I430A-R | 5’-CAGACGGCCTGCACGGCCGTCTTCGCCTCCTCGGAACCGTC-3’ |
| Orco-V428A/V431A-F | 5’-CGCGCAGATCGCGTGCCAACAATGCCAGAAGGCGATGACGATC-3’ |
| Orco-V428A/V431A-R | 5’-CACGCGATCTGCGCGAACGTCTTCGCCTCCTCGGAACCG-3’ |
| Orco-K437E-F | 5’-TGCCAACAATGCCAGGAGGCGATGACGATCTCTGGAGCGAAATTC-3’ |
| Orco-K437E-R | 5’-AGAGATCGTCATCGCCTCCTGGCATTGTTGGCAGACGATCTGC-3’ |
| Orco-L451A/L458A-F | 5’-ATTTGTTCGCTTCGGTGGCGGGCGCTGTTGTCACGTACTTC-3’ |
| Orco-L451A/L458A-R | 5’-CCACCGAAGCGAACAAATCCGCTGAAACATTGAAGAATTTCGCTC-3’ |
| Orco-Y464A/V467A/L468/L471A-F | 5’-CTTCATGGACGACATCCAAGACAAGTAGCTCGAGTCTAGAG-3’ |
| Orco-Y464A/V467A/L468/L471A-R | 5’-GATGTCGTCCATGAAGTCCGTGACAACAGCGCCCAGCAC-3’ |
| B-W33A-F | 5’-GCCTGCTGGGCGCCGCGCCCGGCCACTACCTGGGCGAGGAG-3’ |
| B-W33A-R | 5’-GGTAGTGGCCGGGCGCGGCGCCCAGCAGGCGCAGGCTGGTG-3’ |
| B-W103A-F | 5’-GCTTCAGCCTGACCGCGAACCCCAACTACCACAAGGTGGTG-3’ |
| B-W103A-R | 5’-TGGTAGTTGGGGTTCGCGGTCAGGCTGAAGCCGCGGCTCAG-3’ |
| B-W209A-F | 5’-CATCTGCGTGTTCAACGCGTTCGCCAGCTACATCTGCTGCAG-3’ |
| B-W209A-R | 5’-GCAGATGTAGCTGGCGAACGCGTTGAACACGCAGATGAAGATG-3’ |
| B-W286A-F | 5’-AGCCCCGCCGAGATGGCGCAGGTGACCGACCGCCTGCGCCAG-3’ |
| B-W286A-R | 5’-CAGGCGGTCGGTCACCTGCGCCATCTCGGCGGGGCTGTACAG-3’ |
| B-W383A-F | 5’-GTGTACGAGGTGCCCGCGGAGTACATGGACACCAGCAACCGCAAG-3’ |
| B-W383A-R | 5’-GTGTCCATGTACTCCGCGGGCACCTCGTACACGGCGTCCTTCAG-3’ |
| B-1-43JD-F | 5’-ACGAGCTGTACAAGAGCAAGTACGAGTGCGCCCCCATG-3’ |
| B-1-43JD-R | 5’-CACTCGTACTTGCTCTTGTACAGCTCGTCCATGCCGTGAG-3’ |
